# Stress testing the Centiloid: Precision and variability of PET quantification of amyloid pathology

**DOI:** 10.1002/alz.13883

**Published:** 2024-07-04

**Authors:** Mahnaz Shekari, David Vállez García, Lyduine E. Collij, Daniele Altomare, Fiona Heeman, Hugh Pemberton, Núria Roé Vellvé, Santiago Bullich, Christopher Buckley, Andrew Stephens, Gill Farrar, Giovanni Frisoni, William E. Klunk, Frederik Barkhof, Juan Domingo Gispert, ADNI and the AMYPAD consortium

**Affiliations:** ^1^ Barcelonaβeta Brain Research Center (BBRC) Pasqual Maragall Foundation Barcelona Spain; ^2^ IMIM (Hospital del Mar Medical Research Institute) Barcelona Spain; ^3^ Universitat Pompeu Fabra Barcelona Spain; ^4^ Department of Radiology and Nuclear Medicine Amsterdam UMC, Vrije Universiteit Amsterdam Amsterdam Netherlands; ^5^ Clinical Memory Research Unit Clinical Sciences Malmö Lund University Malmö Sweden; ^6^ Memory Center Department of Rehabilitation and Geriatrics University Hospitals and University of Geneva Genève Switzerland; ^7^ Wallenberg Centre for Molecular and Translational Medicine University of Gothenburg, The University of Gothenburg Gothenburg Sweden; ^8^ Department of Psychiatry and Neurochemistry University of Gothenburg Sahlgrenska University Hospital Gothenburg Sweden; ^9^ GE Healthcare Life Sciences Amersham UK; ^10^ Institute of Neurology and Centre for Medical Image Computing University College London London UK; ^11^ Life Molecular Imaging GmbH Berlin Germany; ^12^ University of Pittsburgh Pittsburgh Pennsylvania USA; ^13^ Centro de Investigación Biomédica en Red Bioingeniería, Biomateriales y Nanomedicina, (CIBER‐BBN) Madrid Spain

**Keywords:** age, Alzheimer's disease, amyloid PET accuracy, biomarker validation, brain atrophy, clinical applications, clinical trials, context of use, diagnosis, disease‐modifying therapies, image harmonization, radiotracers, white matter

## Abstract

**INTRODUCTION:**

Assessing the potential sources of bias and variability of the Centiloid (CL) scale is fundamental for its appropriate clinical application.

**METHODS:**

We included 533 participants from AMYloid imaging to Prevent Alzheimer's Disease (AMYPAD DPMS) and Alzheimer's Disease Neuroimaging Initiative (ADNI) cohorts. Thirty‐two CL pipelines were created using different combinations of reference region (RR), RR and target types, and quantification spaces. Generalized estimating equations stratified by amyloid positivity were used to assess the impact of the quantification pipeline, radiotracer, age, brain atrophy, and harmonization status on CL.

**RESULTS:**

RR selection and RR type impact CL the most, particularly in amyloid‐negative individuals. The standard CL pipeline with the whole cerebellum as RR is robust against brain atrophy and differences in image resolution, with 95% confidence intervals below ± 3.95 CL for amyloid beta positivity cutoffs (CL < 24).

**DISCUSSION:**

The standard CL pipeline is recommended for most scenarios. Confidence intervals should be considered when operationalizing CL cutoffs in clinical and research settings.

**Highlights:**

- We developed a framework for evaluating Centiloid (CL) variability to different factors.
- Reference region selection and delineation had the highest impact on CL values.
- Whole cerebellum (WCB) and whole cerebellum plus brainstem (WCB+BSTM) as reference regions yielded consistent results across tracers.
- The standard CL pipeline is robust against atrophy and image resolution variation.
- Estimated within‐ and between‐pipeline variability (95% confidence interval) in absolute CL units.

## 1 BACKGROUND

Positron emission tomography (PET) imaging enables the measurement of cerebral amyloid beta (Aβ) pathology load and spread, a defining characteristic of Alzheimer's disease (AD). In addition to visual read (VR) assessment, quantification of Aβ PET is used in clinical trials studying anti‐Aβ disease‐modifying therapies; thus, it is being considered for clinical use.^1^

The quantification of amyloid PET is usually performed through the standard uptake value ratio (SUV_r_), the ratio between an amyloid‐avid region of interest (ROI) and a reference region (RR), devoid of specific binding sites. However, SUV_r_ values are not comparable when using dissimilar tracers, ROIs, or image‐processing algorithms (also known as “pipelines”).^2^ To overcome these limitations, the Centiloid (CL) method was proposed to render standard units of Aβ load that is insensitive to these confounders.^3^ The availability of standard units enables the derivation of generalizable CL cutoffs that can be applied in clinical and research settings. For example, CL values below 10–12 are optimal for ruling out the presence of amyloid pathology, whereas CL values over 30 relate to established Aβ pathology.^4^, ^5^, ^6^ Clinical trials of anti‐Aβ disease‐modifying treatments have shown reductions of 65–90 CL.^7^ Notably, the U.S. Food and Drug Administration (FDA) now recognizes the reduction in Aβ load as a surrogate endpoint of drug efficacy.^8^ CL cutoffs have been set a priori to guide dose selection and treatment cessation.^9^ Finally, prevention trials such as the AHEAD 3‐45 recruit cognitively unimpaired participants based on their CL values.^10^ Together these use cases support the CL as a well‐established metric of Aβ with potential clinical application.

However, the robustness of CL cutoffs to image quantification pipeline, age, brain atrophy, and image harmonization has not been well established in sufficiently representative samples. To address this issue, we assessed the sensitivity of this metric, in absolute CL units, to these factors in a typical memory clinic sample. In total, 32 different pipelines were implemented, validated, and assessed. In addition, we quantified the impact of age, brain atrophy, image harmonization, and their interactions with the three approved amyloid tracers. Finally, we provide recommendations for the optimal pipeline design options.

## 2 METHODS

### 2.1 Subjects and Aβ PET image acquisition

A total of 533 participants from the AMYPAD DPMS (Amyloid Imaging to Prevent Alzheimer's Disease – Diagnostic and Patient Management Study) and the ADNI (Alzheimer's Disease Neuroimaging Initiative) were included.

AMYPAD DPMS recruited participants representative of a typical clinical population.^11^ We included all available AMYPAD DPMS participants with valid baseline T1‐weighted 3T magnetic resonance imaging (MRI) and Aβ PET scans acquired with either ^18^F‐Flutemetamol (FMM; Vizamyl) or ^18^F‐Florbetaben (FBB; Neuraceq). To minimize between‐scanner differences, all PET images were harmonized using Hoffman phantom scans to achieve an 8 mm effective image resolution.^12^

To include all approved Aβ PET tracers, we also included ^18^F‐Florbetapir (FBP) PET scans from ADNI with available T1‐weighted MRI. For ADNI participants, isotropic T1‐weighted MRI scans were acquired with 1.5T or 3T scanners. All ADNI PET images were harmonized a priori to an 8 mm effective image resolution.^13^ A total of 867^18^F‐Florbetapir amyloid PET scans were initially available from the ADNI study. However, to prevent overrepresenting FBP tracer in the analysis, a subsample of ADNI scans was selected using SPSS (version 29.0.2.0) case–control matching.^14^ This subsample was chosen to match the age, sex, clinical diagnosis, and prevalence of amyloid positivity between ADNI and AMYPAD DPMS as closely as possible. As a result, a total of 203 cases from ADNI were matched with the AMYPAD DPMS subjects. More details can be found in Table S1.

### 2.2 Aβ PET quantification

Quantification of the PET images was performed following the CL framework and using the respective MRI scans. A detailed description of the CL calibration methodology is provided in Supplementary information. Then, using the standard Statististical Parametric Mapping (SPM) Centiloid pipeline from the Global Alzheimer's Association Interactive Network (GAAIN) Centiloid project website (https://www.gaain.org/centiloid-project) as the reference, we created 32 different variations, all based on SPM12, consisting of all possible combinations of the following design options: reference region (RR: whole cerebellum [WCB], cerebellum gray matter [CGM], pons and whole cerebellum plus brainstem [WCB+BSTM]), RR type (GAAIN and “subject‐based”), cortical target definition (GAAIN and “subject‐based”), and quantification space (Montreal Neurological Institute [MNI] and subject space). All CL pipelines were calibrated for ^18^F‐Flutemetamol, ^18^F‐Florbetaben, and ^18^F‐Florbetapir and validated according to the standard criteria.^3^ Details on RR and cortical target definition, as well as conversion equations and the results of the validation process, can be found in the Supplementary information. Preprocessing steps used for CL quantifications are available on https://github.com/MahnazShekari/Centiloid‐pipeline. For the DPMS, CL values were calculated for both original (un‐harmonized) and harmonized PET scans resulting in 64 measurements per subject. For the ADNI cohort, only harmonized PET scans were available, and 32 CL values were calculated for each scan.

### 2.3 Quality control

Quality control of preprocessing steps and outputs of the pipelines was done visually for all images prior to calculating CL values.

### 2.4 Statistical analysis

Descriptive statistics were calculated for the main demographic variables of the participants grouped by tracer. Parametric or nonparametric group comparison statistics were calculated as appropriate.

Analyses were stratified by amyloid positivity, considered if CL > 24^15^ as calculated with the standard CL pipeline (see Supplementary information).

**RESEARCH IN CONTEXT**

1. **Systematic review**: We reviewed the literature using PubMed. Although there have been several publications on the application of Centiloid (CL) in the research context, the impact of different technical factors, age, and brain atrophy on CL variability has not been well established in sufficiently representative samples.
2. **Interpretation**: Our results indicated that optimal pipeline design is essential for deriving robust CL values under different clinical and research settings. The 95% confidence interval of within‐pipeline differences should be considered when operationalizing CL cutoffs.
3. **Future directions**: Applying our framework to the longitudinal positron emission tomography images to evaluate the impact of technical factors, age, brain atrophy, and harmonization status on different CL pipelines longitudinally.

Generalized estimating equations (GEEs) were used to evaluate the impact of the different pipeline design factors on CL values. Two types of models were computed: “bias models” and a “precision model.” The main bias model included all technical factors to assess their individual impact on CL values and their interaction with tracer. Secondary bias models additionally assessed the impact of age, brain atrophy (both between‐subject factors), and image harmonization (within‐subject), and their interaction with tracer and pipeline design factors on CL.

The main bias model was the following:

(1) $$\begin{array}{ccc} & & CL∼\;\mathrm{Intercept}+\mathrm{Clinical}\_\mathrm{diagnosis}+\mathrm{Tracer}+\mathrm{RR}+\mathrm{RR}\;\mathrm{type}\\ & & \;+\;\mathrm{Space}+\mathrm{Ttype}+\mathrm{Tracer}+\mathrm{Tracer}*\mathrm{RR}+\mathrm{Tracer}*\mathrm{RR}\;\mathrm{type}\\ & & \;+\;\mathrm{Tracer}*\mathrm{Ttype}+\mathrm{Tracer}*\mathrm{Space} \end{array}$$

Pipeline design factors, namely RR selection, RR type, cortical target type (Ttype), and quantification space (Space) were introduced to the model as within‐subject factors, both independently and in interaction with tracer to evaluate possible impact of tracer on CL variability. Tracer and clinical diagnosis were entered into the model as between‐subject factors.

The three secondary “bias models” were as follows:

(2) $$\begin{array}{ccc} & & CL∼\;\mathrm{Intercept}+\mathrm{Clinical}\_\mathrm{diagnosis}+\mathrm{Tracer}+\mathrm{RR}+\mathrm{RR}\;\mathrm{type}\\ & & \;+\;T\;\mathrm{type}+\mathrm{Space}+\mathrm{Age}+\mathrm{Tracer}*\mathrm{RR}+\mathrm{Tracer}*\mathrm{RR}\;\mathrm{type}\\ & & \;+\;\mathrm{Tracer}*T\;\mathrm{type}+\mathrm{Tracer}*\mathrm{Space}+\mathrm{Age}*\mathrm{RR}+\mathrm{Age}*\mathrm{RR}\;\mathrm{type}\\ & & \;+\;\mathrm{Age}*T\;\mathrm{type}+\mathrm{Age}*\mathrm{Space} \end{array}$$

(3) $$\begin{array}{ccc} & & CL∼\;\mathrm{Intercept}+\mathrm{Clinical}\_\mathrm{diagnosis}+\mathrm{Tracer}+\mathrm{Brain}\_\mathrm{Atrophy}\\ & & \;+\;\mathrm{RR}+\mathrm{RR}\;\mathrm{type}+\mathrm{Ttype}+\mathrm{Space}+\mathrm{Tracer}*\mathrm{RR}\\ & & \;+\;\mathrm{Tracer}*\mathrm{RR}\;\mathrm{type}+\mathrm{Tracer}*\mathrm{Ttype}+\mathrm{Tracer}*\mathrm{Space}\\ & & \;+\;\mathrm{Brain}\_\mathrm{Atrophy}*\mathrm{RR}+\mathrm{Brain}\_\mathrm{Atrophy}*\mathrm{RR}\;\mathrm{type}\\ & & \;+\;\mathrm{Brain}\_\mathrm{Atrophy}*\mathrm{Ttype}+\mathrm{Brain}\_\mathrm{Atrophy}*\mathrm{Space} \end{array}$$

(4) $$\begin{array}{ccc} & & CL∼\;\mathrm{Intercept}+\mathrm{Clinical}\_\mathrm{diagnosis}+\mathrm{Tracer}+\mathrm{Image}\_\mathrm{Harmonization}\\ & & \;+\;\mathrm{RR}+\mathrm{RR}\;\mathrm{type}+T\;\mathrm{type}+\mathrm{Space}+\mathrm{Tracer}*\mathrm{RR}+\mathrm{Tracer}*\mathrm{RR}\;\mathrm{type}\\ & & \;+\;\mathrm{Tracer}*T\;\mathrm{type}+\mathrm{Tracer}*\mathrm{Space}+\mathrm{Image}\_\mathrm{Harmonization}*\mathrm{RR}\\ & & \;+\;\mathrm{Image}\_\mathrm{Harmonization}*\mathrm{RRtype}+\mathrm{Image}\_\mathrm{Harmonization}*\mathrm{Ttype}\\ & & \;+\;\mathrm{Image}\_\mathrm{Harmonization}*\mathrm{Space}\; \end{array}$$

Brain atrophy was defined as total GM volume normalized to total intracranial volume (TIV) (Supplementary information).

The “precision model” aimed to establish within‐ and between‐pipeline 95% confidence intervals (95% CIs) of CL values, as follows:

(5) CL∼Intercept+Pipeline

Where “Pipeline” is a within‐subject factor that refers to one specific CL pipeline (Table S2). Between‐pipeline and within‐pipeline variabilities and corresponding 95% CIs were calculated for both the amyloid‐negative and amyloid‐positive groups. Finally, using linear interpolation, 95% CIs were calculated for the range of typical cutoff values for amyloid positivity, ranging from CL = 12^5^, ^6^ to CL = 24,^15^ as well as between 0 and 100 CL.

The main outcome measure of our models is the marginal mean difference (and 95% CIs) in CL units between different levels of the studied within‐subject factors (e.g., the CL differences when using different RRs), which are expected to be zero. Differences below 3 CL were considered as irrelevant as this is the estimated test–retest variability of Aβ PET in CL units.^16^, ^17^ The relative influence of factors is denoted by Wald chi‐square values denoting the relative amount of variability in CL values that is accounted for by each factor (e.g., whether RR is associated with larger CL within‐subject differences than quantification space). Similarly, we compared Wald chi‐square values of a particular factor across main and secondary models to assess whether the variability in the main model can be accounted for by another factor in the secondary model (e.g., whether differences in the RR can be explained by age). The intraclass correlation coefficient (ICC) was calculated for comparing CL values before and after harmonization, and the Bland–Altman plot was used to show the impact of harmonization status on CL values as a function of RR selection. *p*‐values < 0.05 were considered statistically significant. All statistical analyses were performed with SPSS.

## 3 RESULTS

Table 1 shows demographics, clinical diagnosis, and CL values for participants stratified by PET tracer. Figure S1 shows a box‐and‐whiskers plot of the SUVr and CL values for the amyloid‐negative and amyloid‐positive groups for all RRs and tracers.

**TABLE 1:** Demographic information per cohort and tracer.

Demographic					
Cohort	AMYPAD DPMS		ADNI		
Tracer	FMM	FBB	FBP	*p*‐value	Total
*N*	207	123	203	NA	533
Age	70.4 ± 7.0	70.7 ± 7.5	72.1 ± 5.7	0.08	71.1 ± 6.7
Sex (female%)	99 (47.8%)	39 (31.7%)	83 (40.9%)	**0.02**	221 (41.46%)
Clinical status					
CU+SCD	79 (38.2%)	31 (25.2%)	60 (29.6%)	**<0.001**	170 (31.9%)
MCI	90 (43.5%)	44 (35.8%)	104 (51.2%)		238 (44.6%)
Dementia	38 (18.4%)	48 (39%)	39 (19.2%)		125 (23.5%)
Centiloid (Mean ±SD)	36.9 ± 40.7	52.2 ± 45.1	46.84 ± 45.9	**0.001**	45.9 ± 46.0

*Note*: The *p*‐value represents the result of the between‐group comparison for demographic criteria.

Abbreviations: AD, Alzheimer's disease; ADNI, Alzheimer's Disease Neuroimaging Initiative; CU, cognitively unimpaired; FBB, ^18^F‐Florbetaben; FBP, ^18^F‐Florbetapir; FMM, ^18^F‐Flutemetamol; MCI, mild cognitive impairment; NA: Not applicable; SCD, subjective cognitive decline.

Bold values indicate *p*‐values < 0.05.

### 3.1 Main bias model

Table 2 shows the results of the “bias” GEE model for Aβ‐negative and Aβ‐positive scans. Tracer had no statistically significant effect on CL values for either amyloid‐negative or amyloid‐positive groups (Table 2A,B). Consequently, comparable CL marginal means were observed for both groups across tracers. Clinical diagnosis was statistically significant only for the amyloid‐positive group, with higher CL values for more severe clinical stages: subjective cognitive decline (SCD): 64.63 ± 4.10CL; mild cognitive impairment (MCI): 76.29 ± 2.71CL; and AD: 88.04 ± 2.99CL.

**TABLE 2:** Results for the base GEE model for amyloid‐negative and amyloid‐positive groups, respectively (A and B).

Amyloid‐negative group				Amyloid‐positive group			
(A) Bias Model				(B) Bias Model			
	**Type III**				**Type III**		
**Source**	**Wald chi‐square**	**df**	**Sig**.	**Source**	**Wald chi‐square**	**df**	**Sig**.
(Intercept)	2.10	1	0.15	**(Intercept)**	**1568.10**	**1**	**<0.001**
Tracer	1.19	2	0.55	Tracer	0.73	2	0.70
Clinical diagnosis	0.85	2	0.65	**Clinical diagnosis**	**22.26**	**2**	**<0.001**
**Reference region (RR)**	**318.02**	**3**	**<0.001**	**Reference region (RR)**	**102.40**	**3**	**<0.001**
**RR type**	**198.87**	**1**	**<0.001**	**RR type**	**54.62**	**1**	**<0.001**
**Target type**	**59.22**	**1**	**<0.001**	**Target type**	**9.83**	**1**	**0.002**
**Space**	**5.44**	**1**	**0.020**	**Space**	**47.04**	**1**	**<0.001**
Tracer * RR	**224.01**	**6**	**<0.001**	Tracer * RR	**116.78**	**6**	**<0.001**
Tracer * RR type	**13.22**	**2**	**0.001**	Tracer * RR type	**10.53**	**2**	**0.005**
Tracer * Target type	**24.08**	**2**	**<0.001**	Tracer * Target type	**30.34**	**2**	**<0.001**
Tracer * Space	**56.53**	**2**	**<0.001**	Tracer * Space	**86.85**	**2**	**<0.001**

(C) Marginal means comparison			(D) Marginal means comparison		
Tracer	Mean (95% CI)		Tracer	Mean (95% CI)	
FMM	2.40 (−0.37 to 5.16)		FMM	76.91 (71.01 to 82.80)	
FBB	2.77 (−1.92 to 7.46)		FBB	74.06 (67.04 to 81.08)	
FBP	1.11 (−1.89 to 4.12)		FBP	78.01 (72.03 to 83.98)	

RR	Mean (95% CI)	∆CL (95% CI)	RR	Mean (95% CI)	∆CL (95% CI)
WCB	4.70 (1.88 to 7.52)	Reference	WCB	77.85 (74.03 to 81.66)	Reference
CGM	5.53 (2.81 to 8.26)	0.84 (0.19 to 1.48)	CGM	76.26 (72.33 to 80.19)	−1.59 (−2.47 to −0.71)
WCB+BSTM	2.36 (−0.49 to 5.21)	−2.34 (−2.67 to −2.00)	WCB+BSTM	76.87 (73.08 to 80.67)	−0.97 (−1.54 to −0.40)
**Pons**	**−4.22 (−7.50 to −0.94)**	**−8.92 (−10.63 to −7.48)**	**Pons**	**74.32 (70.01 to 78.63)**	**−3.53 (−6.04 to −1.01)**

RR type	Mean (95% CI)	∆CL (95% CI)	RR type	Mean (95% CI)	∆CL (95% CI)
GAAIN	4.31 (1.43 to 7.20)	Reference	GAAIN	77.66 (73.90 to 81.43)	Reference
**Subject‐based**	**−0.13 (−2.94 to 2.68)**	**−4.44 (−5.06 to −3.82)**	Subject‐based	74.98 (71.16 to 78.80)	−2.68 (−3.39 to −1.97)

Target type	Mean (95% CI)	∆CL (95% CI)	Target type	Mean (95% CI)	∆CL (95% CI)
GAAIN	0.36 (−2.49 to 3.22)	Reference	GAAIN	75.76 (71.98 to 79.54)	Reference
**Subject‐based**	**3.82 (0.95 to 6.70)**	**3.46 (2.58 to 4.34)**	Subject‐based	76.88 (73.08 to 80.70)	1.13 (0.42 to 1.83)

Space	Mean (95% CI)	∆CL (95% CI)	Space	Mean (95% CI)	∆CL (95% CI)
MNI	0.88 (−1.37 to 3.12)	Reference	MNI	77.54 (73.64 to 81.45)	Reference
Native	3.31 (−0.31 to 6.93)	2.44 (0.39 to 4.48)	Native	75.11 (71.43 to 78.79)	−2.43 (−3.13 to −1.47)

*Notes*: The technical factors demonstrating statistical significance are highlighted in red. Marginal means and 95% confidence intervals for tracer, RR selection, RR type, target type, and quantification space for amyloid‐negative (C) and amyloid‐positive (D) groups. The last columns of (C) and (D) show differences between marginal means with respect to the reference technical design (RR: WCB; RR type: GAAIN; Target type: GAAIN; Space: MNI). Marginal mean differences exceeding ± 3CL are indicated in red.

Abbreviations: CGM, cerebellum gray matter; CL, Centiloid; FBB, 18F‐Florbetaben; FBP, 18F‐Florbetapir; FMM, 18F‐Flutemetamol; RR, Reference Region; WCB, whole cerebellum; WCB+BSTM, whole cerebellum plus brainstem.

Bold values indicate *p*‐values < 0.05.

**FIGURE 1 alz13883-fig-0001:**
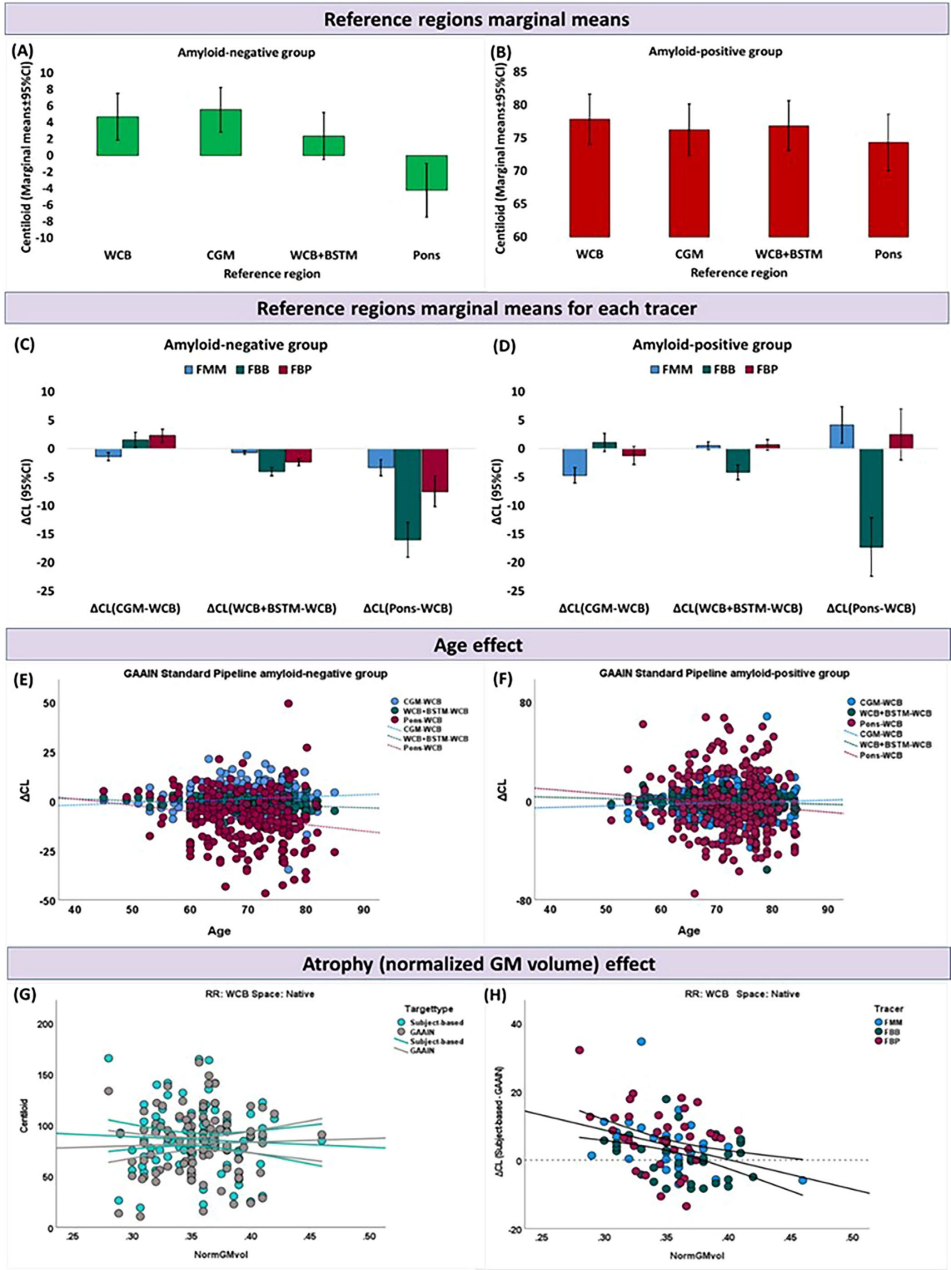
The marginal means of Centiloid (CL) values for each reference region for amyloid‐negative (A) and amyloid‐positive (B) group. C and D show the difference in marginal means for each Reference Region (RR) versus Whole Cerebellum (WCB) per tracers for amyloid‐negative and amyloid‐positive groups, respectively. Differences in the CL between WCB and Cerebellar Gray Matter (CGM), Whole Cerebellum plus Brainstem (WCB+BSTM), and pons as a function of age for amyloid‐negative and amyloid‐positive groups, respectively (E, F). G shows CL (95% CI) values extracted from two different pipelines using predefined GAAIN cortical target ROI and subject‐based cortical target Region of Interest (ROI). (H) Scatter plot showing the differences (95% CI) in the calculated CL when using subject‐based cortical target versus predefined GAAIN cortical target ROI as a function of global brain atrophy (normalized gray matter volume) for participants diagnosed with dementia, and with positive amyloid status. CGM, cerebellum gray matter; CL, Centiloid; FBB, ^18^F‐Florbetaben; FBP, ^18^F‐Florbetapir; FMM, ^18^F‐Flutemetamol; ROI, region of interest; RR, reference region; WCB, whole cerebellum; WCB+BSTM, whole cerebellum plus brainstem.

Regarding pipeline design factors, RR, its interaction with tracer, and RR type accounted for most of the CL variability in both groups, as reflected by higher Wald chi‐square values. CL marginal means for RR selection, RR type, target type, and quantification space stratified by amyloid status are shown in Table 2C,D. In the amyloid‐negative group, using the pons as RR resulted in the lowest mean CL values (Figure 1A). A similar behavior was observed in the positive group, albeit with smaller effects. Figure 1C shows a similar behavior across all 3 tracers (∆CL_Pons‐WCB_: FMM = −3.13 ± 0.71; FBB = −15.97 ± 1.56; FBP = −7.48 ± 1.38) in the amyloid‐negative group. In contrast, for the for amyloid‐positive group, between‐tracer differences were prominent, with smaller effects for FMM and FBP (∆CL_Pons‐WCB_: FMM = 4.19 ± 1.65; FBP = 2.50 ± 2.28). Subject‐based RR rendered lower CL values than GAAIN RRs (∆CL: amyloid‐negative = −4.44 ± 0.31 and amyloid‐positive = −2.68 ± 0.36]. Using subject‐based cortical target ROI yielded slightly higher CL values (∆CL: amyloid‐negative = 3.46 ± 0.45, amyloid‐positive = 1.13 ± 0.36). Quantification space had a minor impact on CL (∆CL∼2.43) for both groups (Table 2C,D).

### 3.2 Secondary bias models

#### 3.2.1 Age

The main effect of age was not associated with the CL values in either of the groups (Table S3). However, its interaction with the choice of RR significantly reduced the Wald chi‐square values for RR for both groups, indicating that most of the variability associated with RR can be accounted for by age‐related effects. Indeed, in the amyloid‐negative group, significant negative correlations were found between age and CL differences derived from different RRs (∆CL(WCB‐WCB+BSTM): [rho = −0.22, *p* < 0.001] or ∆CL(WCB‐Pons): [rho = −0.20, *p* = 0.002]) (Figure 1E). In the amyloid‐positive group, a significant positive correlation was observed between ∆CL(CGM‐WCB) and age (rho = 0.16, *p* = 0.01), whereas an inverse association between age and ∆CL was observed when using WCB+BSTM versus WCB as RR (rho = −0.12, *p* = 0.04), (Figure 1F).

#### 3.2.2 Brain atrophy

Brain atrophy was not significantly associated with CL values in either the amyloid‐negative or amyloid‐positive group (Table S4). In this model, a statistically significant interaction between atrophy and technical factors were observed for RR, RR type, and target type, respectively. Figure 1G shows that there is no significant correlation between CL values calculated using a predefined GAAIN cortical target (*rh* = −0.02, *p* = 0.87) versus subject‐based cortical target (*rh* = 0.01, *p* = 0.90) as a function of brain atrophy. However, the subject‐based cortical target was compared to the GAAIN cortical target for amyloid‐positive participants diagnosed with dementia, a mean difference of ΔCL = 3.99 ± 8.21 was observed (Figure 1H). In addition, a negative correlation was observed between brain atrophy and differences in CL when using subject‐based cortical target versus GAAIN cortical target (*r* = −0.35; *p* < 0.001), confirming that in the presence of atrophy, the use of GAAIN cortical ROI resulted in lower Aβ load estimates by ∼10CL in the subjects with the highest atrophy.

#### 3.2.3 Image harmonization

The choice of RR and target type had a significant effect on CL values depending on harmonization status, whereas quantification space did not (Table S5). Notably, the use of pons as the RR had a substantial impact on the marginal means after harmonization for both the amyloid‐negative (ΔCL = 7.25 ± 0.34) and amyloid‐positive (ΔCL = 4.58 ± 0.19) groups. Furthermore, when CGM was used as the RR, harmonization of PET images resulted in a difference of ΔCL = −8.29 ± 0.31 specifically within the amyloid‐positive group (Table 3). Subject‐based cortical target ROI demonstrated greater sensitivity to harmonization, leading to a difference of ΔCL = 3.62 ± 0.54 for the amyloid‐negative group and ΔCL = −3.63 ± 0.24 for the amyloid‐positive group.

**TABLE 3 alz13883-tbl-0003:** Pairwise comparisons of the marginal means and 95% confidence interval for technical factors including reference region (RR), target type, RR type, and quantification space before and after harmonization.

Pairwise comparisons			
**(A) Amyloid‐negative group**		**(B) Amyloid‐positive group**	
	Estimates		Estimates
Harm_Status	∆CL (Harmonized‐Original) (95% CI)	Harm_Status	∆CL (Harmonized‐Original) (95% CI)
Harmonized‐Original	2.88 (1.84 to 3.91)	Harmonized‐Original	−1.90 (−2.25 to −1.55)
Reference region (RR)	∆CL (Harmonized‐Original) (95% CI)	Reference region (RR)	∆CL (Harmonized‐Original) (95% CI)
WCB	2.24 (3.38 to 1.10)	WCB	−2.79 (−3.18 to −2.42)
CGM	−1.70 (−3.11 to −0.29)	**CGM**	**−8.30 (−8.92 to −7.68)**
**WCB+BSTM**	**3.45 (2.44 to 4.47)**	WCB+BSTM	−1.10 (−1.44 to −0.76)
**Pons**	**7.52 (6.84 to 8.20)**	**Pons**	**4.59 (4.21 to 4.96)**
Target type	∆CL (Harmonized‐Original) (95% CI)	Targettype	∆CL (Harmonized‐Original) (95% CI)
**Subject‐based**	**3.62 (2.55 to 4.68)**	**Subject‐based**	**−3.63 (−4.16 to −3.10)**
GAAIN	2.14 (1.12 to 3.16)	GAAIN	2.14 (1.12 to 3.16)
RR type	∆CL (Harmonized‐Original) (95% CI)	RR type	∆CL (Harmonized‐Original) (95% CI)
**Subject‐based**	**3.01 (2.17 to 3.85)**	Subject‐based	−2.02 (−2.38 to −1.67)
GAAIN	2.75 (1.52 to 3.98)	GAAIN	−1.77 (−2.14 to −1.41)
Space	∆CL (Harmonized‐Original) (95% CI)	Space	∆CL (Harmonized‐Original) (95% CI)
**Native**	**3.31 (3.02 to 3.60)**	Native	−1.89 (−2.24 to −1.54)
MNI	2.45 (0.56 to 4.34)	MNI	−1.91 (−2.27 to −1.55)

Abbreviations: CGM, cerebellum gray matter; RR, reference region; WCB, whole cerebellum; WCB+BSTM, whole cerebellum plus brainstem.

Bold values indicate *p*‐values <0.05.

**FIGURE 2 alz13883-fig-0002:**
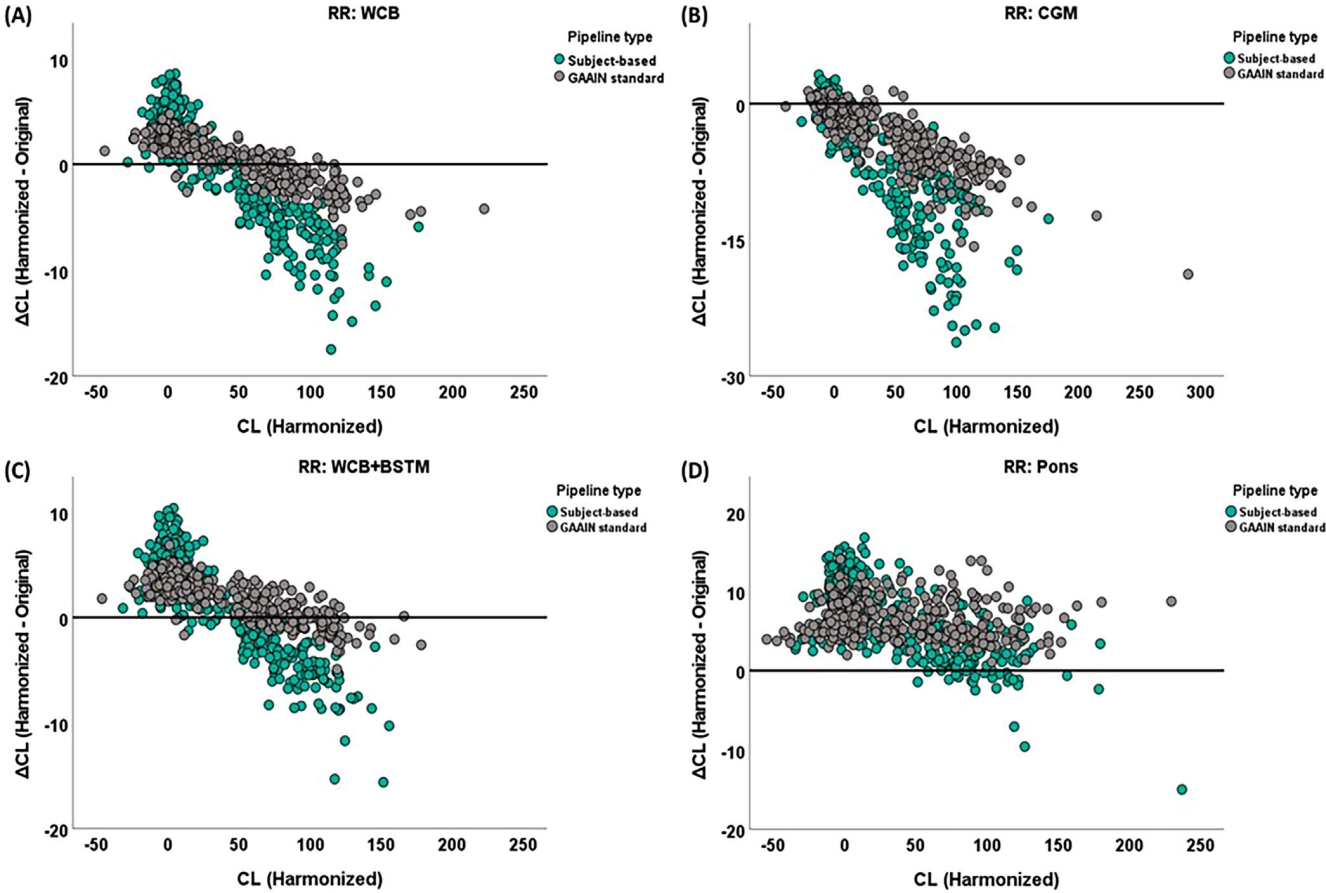
Bland–Altman plots show the difference in the measured Centiloid (CL) between harmonized and original positron emission tomography (PET) images when using (A) whole cerebellum (WCB); (B) cerebellum gray matter (CGM); (C) whole cerebellum plus brainstem (WCB+BSTM); and (D) pons as reference region. It should be noted that all CL values were calculated using predefined GAAIN reference regions and GAAIN cortical target, in MNI space. All graphs include two pipeline types. Gray circles present GAAIN standard pipeline when predefined GAAIN RRs and cortical target ROI are used, and quantification has been done in MNI space. Green circles represent subject‐based pipelines, which have been defined based on subject‐based reference regions and cortical target ROI, in native space. ROI, region of interest; RR, reference region.

The ICC of CL values before and after harmonization was over 0.97 (*p* < 0.001) for all pipelines. Figure 2 shows Bland–Altman plots corresponding to differences in the CL values before and after harmonization using standard GAAIN pipeline and subject‐based pipeline per RR. For GAAIN standard pipelines, using WCB and WCB+BSTM as RRs, yielded the highest agreement between harmonized and original CL values (WCB = 0.47 ± 2.18 [95% CI: 0.24 to 0.71] and WCB+BSTM = 1.80 ± 1.93 [95% CI: 1.59 to 2.01]). In contrast, using CGM and pons as RRs resulted in a difference of ∆CL = −3.58 ± 3.43 (95% CI: −3.95 to −3.21) and ∆CL = 6.49 ± 2.43 (95% CI: 6.23 to 6.76) between harmonized and original CL values, respectively. Using subject‐based cortical target and subject‐based RRs resulted in higher CL differences before and after harmonization (∆CL [95% CI]: WCB = −0.80 ± 5.22 [95% CI: −1.36 to −0.23]; CGM = −6.17 ± 6.55 [95% CI: −6.88 to −5.46]; WCB+BSTM = 0.78 ± 4.95 [95% CI: 0.25 to 1.32]; pons = 5.98 ± 4.59 [95% CI: 5.48 to 6.48]).[Fig alz13883-fig-0001]

### 3.3 Precision model

For the GAAIN standard pipeline, 95% CI of within‐pipeline variability was ± 2.70CL for the amyloid‐negative group and ± 7.43CL for the amyloid‐positive group. Because the within‐pipeline 95% CI was dependent on the CL value, we estimated the 95% CI for two commonly used CL cutoffs with linear interpolation. For the cutoff to rule out the absence of amyloid pathology (CL = 12), the estimated within‐pipeline 95% CI was ± 3.22CL and was ± 3.95CL for the cutoff corresponding to VR positivity (CL = 24) (Supplementary information). The 95% CIs for between‐pipeline variability were ± 6.47CL and ± 4.68CL for the amyloid‐negative and amyloid‐positive groups, respectively (Table S6).[Table alz13883-tbl-0003], [Fig alz13883-fig-0002]

## 4 DISCUSSION

In this study, we assessed the variability of the CL metric to common technical quantification factors and their interactions with the Aβ PET tracer, as well as its sensitivity to the effects of age, brain atrophy, and resolution of the PET scans. Our approach allowed the estimation of the uncertainly and the bias introduced by these factors in absolute CL units with the aim of providing CIs to reference CL cutoff values for clinical and research use. Our results confirmed that the standard CL pipeline, using either WCB or WCB+BSTM as RRs, is robust against all studied factors. It is notable that the effect of the tracer on CL values was not significant. Subject‐based delineation of the target region allowed for increased robustness to brain atrophy, but just when properly harmonized PET scans are available or in single‐center studies. Within‐pipeline variability (95% CI) ranged from ± 3.22 to ± 3.95 CL for the range of useful CL cutoff values for concluding the presence of abnormal Aβ deposition (12–24 CL). We advocate that these CIs are taken into consideration for the operationalization of CL cutoff values in research settings and potential future clinical use.

With respect to technical factors, the choice of the RR had the greatest impact on CL values. Of interest, there was an association between CL values and the percentage of white matter in the RR, suggesting that the observed differences are driven by factors affecting white matter uptake. Because age has been described to be a strong factor affecting white matter uptake with Aβ PET tracers,^18^, ^19^ we confirmed the effect of age in a secondary model. It is important to note that the pons, being primarily composed of white matter, showed the highest discrepancy with age and, therefore, is not optimal for CL scaling. Supplementary analyses showed that the association with age of the pons/WCB ratio, an indicator of white matter uptake, differs significantly by the tracer (Figure S2). It is noteworthy that the same pattern was observed for the elderly individual in the GAAIN data set (Figure S3). Observed age‐related white matter uptake is not driven directly by the average age of the young control group, but by the relative composition of elderly individuals who are amyloid negative for each tracer's reference data set, which may introduce some extent of bias in the estimation of the intercept of the conversion equation for the pons. The extent of bias can also vary by the tracer as a function, not only of the percentage of elderly negative individuals in the reference data sets, but also by the differential effect of age on white matter uptake for each of the tracers. The second most important technical factor is the way the RR was defined with the GAAIN ROIs rendering higher CL values. This may reflect the different composition of gray and white matter depending on how the RR is defined. In turn, target region definition was associated with differences of 3.46CL (95% CI: 2.58 to 4.34) for the amyloid‐negative and 1.13CL (95% CI: 0.42 to 1.83) for the amyloid‐positive group. On top of the presence of white matter in the GAAIN cortical ROI, in this case, differences might stem from the better delineation of individual cortical morphology in subject‐based cortical target ROI.

For a CL pipeline to be applicable in the clinical setting in AD, it should be robust to the presence of brain atrophy. Overall, global brain atrophy did not significantly impact CL measurements. Still, a notable difference of ΔCL = 3.99 ± 8.21 was observed between CL values derived using subject‐based versus predefined GAAIN cortical target ROIs in amyloid‐positive participants diagnosed with dementia, reaching an average of 10CL in those showing the largest extent of atrophy. Note that these participants have an average CL of 90 and, therefore, this difference is not expected to be relevant in practice.

In multicenter studies lacking harmonized brain PET scans, choosing an appropriate pipeline design that is minimally influenced by differences in image resolution is of the utmost importance. Our results suggest that the standard GAAIN pipeline with WCB or WCB+BSTM is robust against differences in image resolution and is, therefore, recommended in this scenario. On the other hand, when using CGM or pons as RR, the CL pipeline became highly sensitive to harmonization status. Because these two RRs consist primarily of gray matter or white matter, they are more prone to signal contamination (“spill‐in”) caused by image resolution variations; therefore they are not suitable RRs for CL quantification in multicenter studies if not accompanied by proper image harmonization procedures. A subject‐based cortical target ROI rendered CL values more sensitive to the harmonization status, which is attributable to the spill‐in effect from white matter uptake to the cortical target.

In this study, we selected ^18^F‐Florbetapir scans in ADNI to match the pooled ^18^F‐Flutemetamol and ^18^F‐Florbetaben subsamples in AMYPAD‐DPMS, to obtain balanced estimates of the studied effects across the three clinically approved ^18^F‐based amyloid PET tracers. Additional analyses were performed to assess the sensitivity of our results to using different statistical models (repeated‐measures analysis of variance [ANOVA], rather than GEEs), analyzing the sample without stratification by amyloid status and stratified by tracer. The results of these sensitivity analyses are not included in the article for the sake of brevity and to confirm the robustness and generalizability of the main analyses. We also calculated the analyses including all available scans from ADNI, but the main estimates leaned to overrepresent the effects of ^18^F‐Florbetapir and underrepresent those of ^18^F‐Flutemetamol.

Our results are in line with the existing literature on the topic. Su et al. explored the capacity of the CL transform to bring several amyloid PET measurements into a common scale, with a focus on the impact on cutoff values.^20^ The observed between‐pipeline 95% CI for the amyloid‐negative group in our work (± 6.47CL) encompasses the variation in CL cutoffs for 95% CI specificity in Su et al. when using different analytic approaches (5.7 to 11.9CL). Bourgeat et al. shows that CapAIBL, a PET‐only quantification method, renders robust and comparable CL estimates than the standard CL pipeline.^21^ They showed that compared to the standard GAAIN pipeline, CapAIBL produced CL values with a negligible bias for PiB, ^18^F‐Florbetapir, and ^18^F‐Flutemetamol. However, larger biases were found for Florbetaben (−14%), which was attributed mostly to the use of different scanners between the calibration scans and the ones used in the study. In this regard, we observed that ^18^F‐Florbetaben was the tracer most sensitive to the effect of age. Finally, it is important to note that alternative ways of estimating the CL transformation have been proposed. Schwarz et al proposed using Deming regression, which accounts for error on both axes, and performing separate regressions for differences in acquisition and analysis methods, rather than direct single‐regression approach.^22^ Still, their results show that these two alternative approaches had performance very similar to that of the standard CL method and within the recommended tolerance thresholds.

Based on the results of these analyses, we propose the following practical recommendations:

1. Using WCB or WCB+BSTM as RR provides comparable results across different tracers and is robust to differences in image resolution.
2. The GAAIN cortical target is robust to the presence of brain atrophy and less sensitive to differences in image resolution in multicenter studies.
3. In the case of a single scanner or available harmonized PET scans, the subject‐based CL pipeline (RR: subject‐based WCB; Target type: subject‐based; Space: Native) can be preferred. This pipeline allows for modeling atrophy in the subject‐based cortical target ROI, provides better RR delineations, and allows quantification of PET images in the native space.
4. Whenever possible, use the same CL pipeline when pooling CL values or in longitudinal studies to minimize CL variability. Consistent pipelines help to avoid incorporating biases that may arise from different pipeline designs, and it provides more reliable results.
5. It is recommended that 95% CI of within‐pipeline differences be incorporated to improve the precision of CL cutoffs for different research purposes.

In this study, we did not have access to a head‐to‐head data set for directly comparing the three different amyloid tracers. Therefore, the finding of no significant main effect of tracer does not necessarily mean there is no difference in the ability of these tracers to quantify amyloid load with CLs. However, independent studies carried out by our group indicate that the CL transform can accurately account for tracer differences.^23^ In addition, our estimation of the between‐pipeline variability of ± 4.68 to ± 6.47 is likely underestimated because all evaluated pipelines were implemented with SPM12 image‐processing pipelines. Nevertheless, previous studies assessing the variability across several commercial CL quantification tools rendered similar results.^24^ It is important to note that longitudinal PET images were not available for the analyses and that further research is necessary to evaluate the performance of different CL pipelines longitudinally. Despite the limitations, our study contributes important information regarding the performance of various CL pipelines and their applicability in different scenarios.

The standard CL method is robust against image resolution differences and the presence of atrophy when using WCB or WCB+BSTM as RR. Subject‐based delineation of the target region allowed for even increased robustness to brain atrophy, but just in when properly harmonized PET scans are available or in single‐center studies. Within‐pipeline variability ranged from ± 3.22 to ± 3.95CL across the range of proposed cutoffs for Aβ abnormality (12–24CL). We recommend considering these CIs to account for the precision of CL values when operationalizing CL‐based cutoff values in a clinical setting or research studies.

The findings of this study have been incorporated into the Biomarker Qualification Opinion (BQO) issued by the European Medicines Agency (EMA).^25^

## CONFLICT OF INTEREST STATEMENT

Dr. Buckley, Dr. Farrar, and Dr. Pemberton are employees of GE Healthcare. Dr. Bullich, Dr. Stephens, and Dr. Vellvé are employees of Life Molecular Imaging GmbH. Dr. Frisoni has received funding through the Private Foundation of Geneva University Hospitals from: A.P.R.A. – Association Suisse pour la Recherche sur la Maladie d'Alzheimer, Genève; Fondation Segré, Genève; Race Against Dementia Foundation, London, UK; Fondation Child Care, Genève; Fondation Edmond J. Safra, Genève; Fondation Minkoff, Genève; Fondazione Agusta, Lugano; McCall Macbain Foundation, Canada; Nicole et René Keller, Genève; Fondation AETAS, Genève. He has received funding through the University of Geneva or Geneva University Hospitals: for IISSs from ROCHE Pharmaceuticals, OM Pharma, EISAI Pharmaceuticals, Biogen Pharmaceuticals, and Novo Nordisk; for competitive research projects from: H2020, Innovative Medicines Initiative (IMI), IMI2, Swiss National Science Foundation, and VELUX Foundation; for consulting from: Biogen, Diadem, Novo Nordisk, and Roche; for honoraria for lectures, presentations, speakers bureaus, manuscript writing, or educational events from: Biogen, Roche, Novo Nordisk, and GE HealthCare. Dr. Altomare received funding by the Fondation Recherche Alzheimer and the Swiss National Science Foundation (project CRSK‐3_196354/1). Dr. Barkhof is a steering committee and iDMC member of studies by Biogen, Merck, Roche, and EISAI. He is a consultant to Roche, Biogen, Merck, IXICO, Jansen, and Combinostics. He has research agreements with Novartis, Merck, Biogen, GE, and Roche and is co‐founder of Queen Square Analytics Ltd. His research is sponsored by the NIHR‐UCLH Biomedical Research Centre, UK MS Society, MAGNIMS‐ECTRIMS, EC‐H2020, EC‐JU (IMI), and EPSRC. Dr. Gispert has received research support from GE Healthcare, Roche Diagnostics, F. Hoffmann – La Roche, speaker's/consultant fees from Biogen, Esteve, Roche Diagnostics and Philips, and served in the Molecular Neuroimaging Scientific Advisory Board of Prothena Biosciences. In addition, he held a “Ramón y Cajal” fellowship (RYC‐2013‐13054), has received research support from the EU/EFPIA Innovative Medicines Initiative Joint Undertaking AMYPAD grant agreement no 115952, and from Ministerio de Ciencia y Universidades (grant agreement RTI2018‐102261). JDG is now a full‐time employee of AstraZeneca. GE Healthcare holds a license agreement with the University of Pittsburgh based on the PiB PET technology related to this manuscript. Dr. Klunk is a co‐inventor of PiB and, as such, has a financial interest in this license agreement. GE Healthcare provided no grant support for this study and had no role in the design or interpretation of results or preparation of this manuscript. The remaining authors declare that the research was conducted in the absence of any commercial or financial relationships that could be construed as a potential conflict of interest. Author disclosures are available in the Supporting information.

## Supporting information

Supporting Information

Supporting Information

Supporting Information
