# Supplementary material for: Stress testing the Centiloid: Precision and variability of PET quantification of amyloid pathology
Source: Alzheimers Dement. 2024 Jul 4;20(8):5102–13. doi: 10.1002/alz.13883 (PMC11350134; doi:10.1002/alz.13883)
Supplement: Supplementary file 1 — Supporting Information [file ALZ-20-5102-s003.docx]

***Stress testing the Centiloid: Precision and variability of PET quantification of amyloid pathology***

Supplement: Centiloid pipeline design and additional analysis

Mahnaz Shekari^1,2,3^, David Vállez García^4^, Lyduine E. Collij^4,5^, Daniele Altomare^6^, Fiona Heeman^4,7,8^, Hugh Pemberton^9,10^, Núria Roé Vellvé^11^, Santiago Bullich^11^, Christopher Buckley^9^, Andrew Stephens^11^, Gill Farrar^9^, Giovanni Frisoni^6^, William E. Klunk^12^, Frederik Barkhof^4,10^, Juan Domingo Gispert^1,2,13^, *On behalf of ADNI and the AMYPAD consortium^14,15^*.

^1^Barcelonaβeta Brain Research Center (BBRC), Pasqual Maragall Foundation. Barcelona, Spain

^2^IMIM (Hospital del Mar Medical Research Institute), Barcelona, Spain

^3^Universitat Pompeu Fabra, Barcelona, Spain

^4^Amsterdam UMC, Vrije Universiteit Amsterdam, Department of Radiology and Nuclear Medicine, De Boelelaan 1117, Amsterdam, Netherlands &

^5^Clinical Memory Research Unit, Clinical Sciences Malmö, Lund University, Lund, Sweden

^6^Memory Center, Department of Rehabilitation and Geriatrics, University Hospitals and University of Geneva Geneva, Switzerland.

^7^Wallenberg Centre for Molecular and Translational Medicine, University of Gothenburg, Sweden

^8^Department of Psychiatry and Neurochemistry, University of Gothenburg, Sahlgrenska University Hospital, Gothenburg, Sweden

^9^ GE Healthcare Life Sciences, Amersham, United Kingdom

^10^Institute of Neurology and Centre for Medical Image Computing, University College London, UK

^11^Life Molecular Imaging GmbH, Berlin, Germany

^12^University of Pittsburgh, Pittsburgh, Pennsylvania, USA.

^13^Centro de Investigación Biomédica en Red Bioingeniería, Biomateriales y Nanomedicina, (CIBER-BBN), Barcelona, Spain

^14^Alzheimer's Disease Neuroimaging Initiative: ADNI

^15^Amyloid Imaging to prevent Alzheimer’s dementia, an IMI/EU funded Project (grant agreement No 115952)

**Complementary analysis for evaluating the age effects**

Figure_s1 shows the calculated SUVr and Centiloid using standard Centiloid pipeline (i.e. Space: MNI; predefined GAAIN cortical target) across different predefined GAAIN reference regions For AMYPAD data, stratified by amyloid status. In Figure_s1 (B), the CL transform eliminates the main differences across tracers and reference regions, but some residual variability remains. Such a residual bias could result from several non-excluding factors, including the pons being prone to segmentation errors due to its small size and problematical spatial normalization (e.g. see Lilja et al. ^1^ among other factors (presence of lesions, etc…).


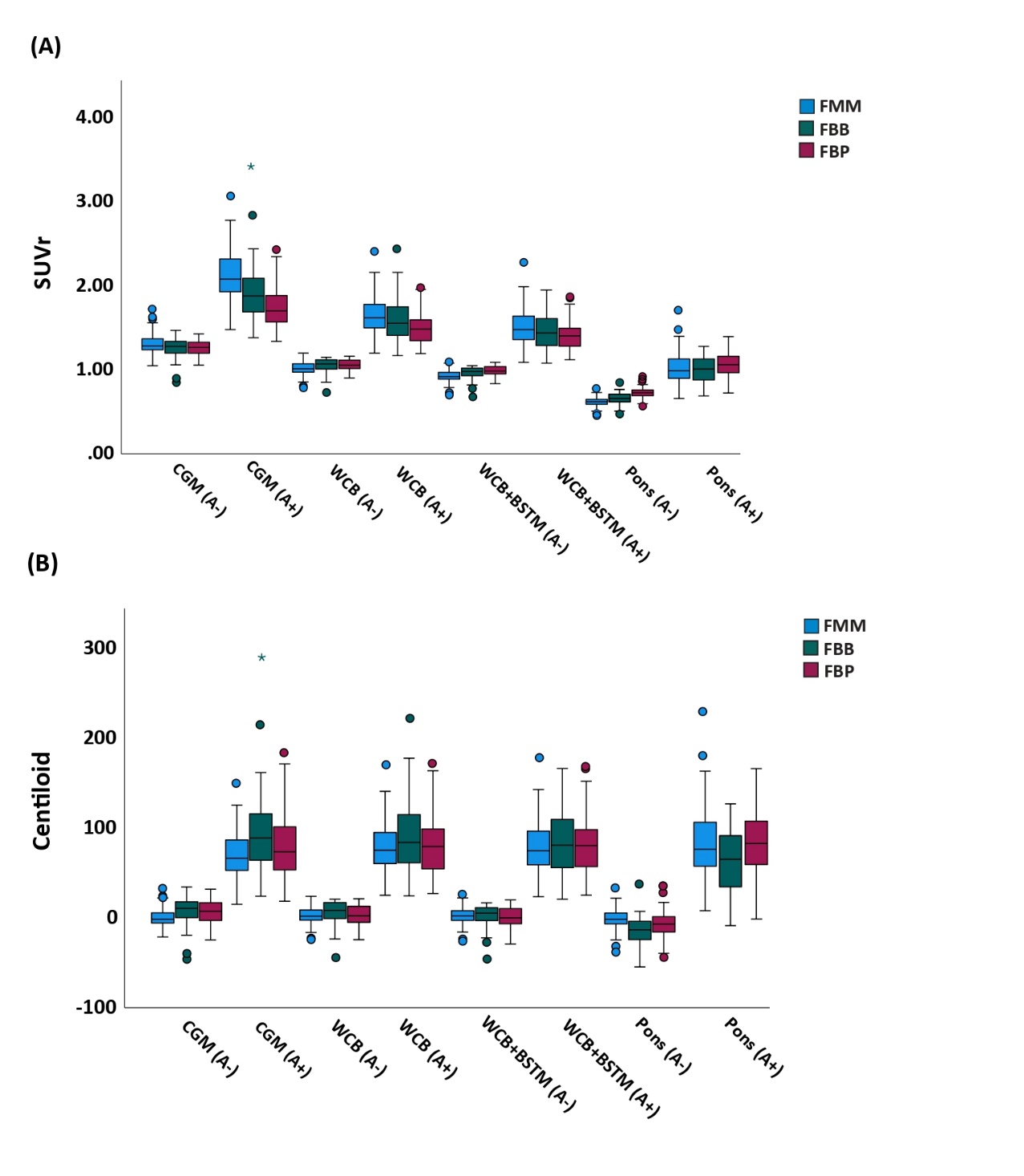


Figure_s1. Calculated SUVr (Panel A) and Centiloid (Panel B) using predefined GAAIN cortical target, reference regions, in MNI space for FMM, FBB, and FBP stratified by amyloid status.


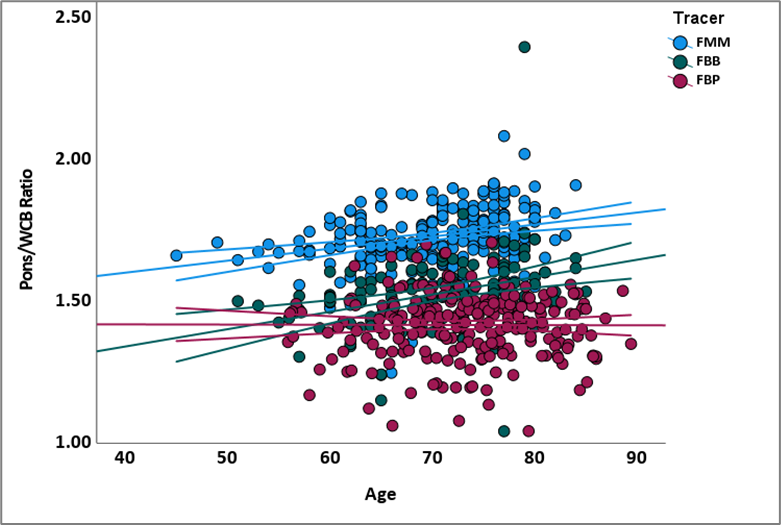


Figure_s2. Associations of the age with the uptake ratio between the pons and the Whole Cerebellum stratified by tracer.

Figure_s2 shows that the tracers represent differential associations of the pons/WC ratio with age, denoting a differential age-related white matter uptake pattern. In this regard, while the ^18^F-Florbetapir pons/WCB is stable with age (r=-0.004; 95%CI: [-0.131, 0.123]; p=0.951), the white matter uptake significantly increases with age for ^18^F-Flutemetamol (r=0.288; 95%CI: [0.164, 0.403]; p<0.001) and, even more, for ^18^F-Florbetaben (r=0.332; 95%CI: [0.165, 0.481]; p<0.001).

We reproduced the previous graph (Figure_s2) for the GAAIN reference dataset, where we calculated the ratio of activity in the Pons to that in the whole cerebellum (WCB) and plotted this ratio against the age in the elderly group (i.e. those who are not Young Controls and which include both amyloid positive [n=66; 28 FMM; 17 FBB; 21 FBP] and amyloid negative [n=41; 21 FMM; 8 FBB; 12 FBP] scans). Figure_s3 shows a positive association between age and Pons/WCB uptake ratio for all the tracers. It is noteworthy that this association exhibits a similar pattern to what we observed in both the AMYPAD DPMS and ADNI datasets, thereby reaffirming the influence of age on white matter uptake in individuals of the GAAIN database of a similar age range than in the samples in our study – ages that would be considered for amyloid imaging in clinical settings.


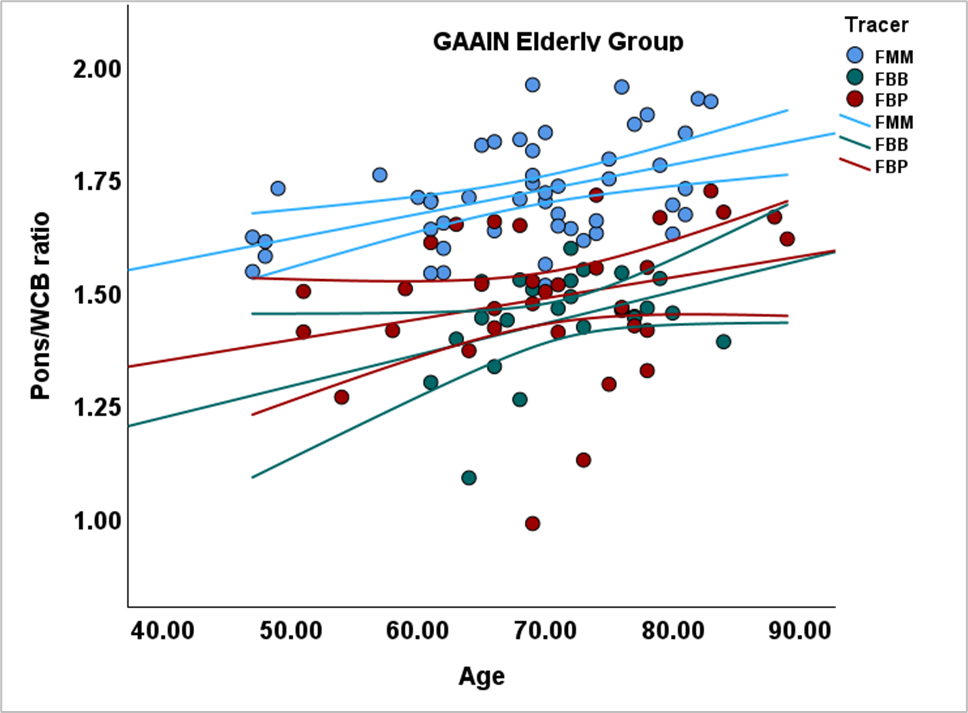


Figure_s3. Associations of age and the ratio of the tracer uptake in pons to Whole Cerebellum for elderly groups in the reference GAAIN dataset; stratified by tracer.

**Case-control matching**

Case-control matching was conducted using SPSS to align age, sex, clinical diagnosis, and amyloid positivity prevalence as closely as possible between ADNI and AMYPAD DPMS participants. Notably, no statistically significant differences were observed in sex, clinical diagnosis, and amyloid positivity between the ADNI subsample and AMYPAD DPMS participants (Table_s1). However, there was a statistically significant difference in age between the two groups (p=0.02), likely attributable to the older age of the ADNI participants compared to those in the AMYPAD DPMS (Figure_s4).

| **Demographic** | | | | | |
| --- | --- | --- | --- | --- | --- |
| **Cohort** | | **AMYPAD DPMS** | **ADNI** | **P-value** | **Total** |
| N | | 330 | 203 | NA | 533 |
| Age | | 70.57±7.24 | 72.1±5.7 | **0.02** | 71.1±6.7 |
| Sex (Female%) | | 138 (41.81%) | 83 (40.9%) | 0.83 | 221(41.46%) |
| Clinical status | CU+SCD | 110 (33.33%) | 60 (29.6%) | 0.71 | 170 (31.9%) |
|  | MCI | 134 (40.60%) | 104 (51.2%) |  | 238 (44.6%) |
|  | Dementia | 86 (26.06%) | 39 (19.2%) |  | 125 (23.5%) |
| Centiloid (Mean±SD) | | 46.21±46.48 | 46.84±45.9 | 0.78 | 45.9±46.0 |

Table_s1. Demographic information per cohort. The p-value represents the result of the between-group comparison for demographic criteria. It should be noted that AMYPAD DPMS includes amyloid PET scans acquired using ^18^F-Flutemetamol or ^18^F-Florbetaben tracers, and ADNI includes amyloid PET scans acquired using ^18^F-Florbetapir tracer. CU: Cognitively unimpaired; SCD: Subjective cognitive decline; MCI: Mild cognitive impairment; NA: Not applicable.


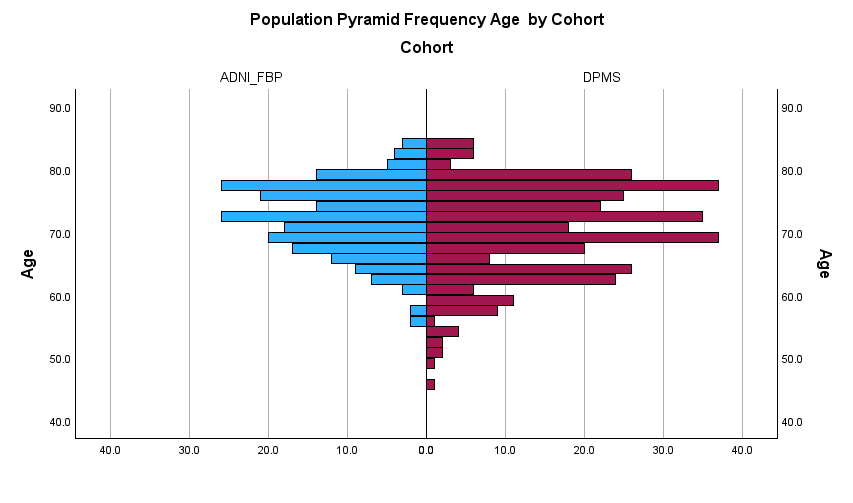


Figure_s4. Age-histograms of ADNI-subsample and AMYPAD DPMS participants.

**Precision model**

Table_s2 shows the design of each pipeline and its corresponding number that was included in the precision model as “pipeline number”. It should be noted that only pipelines using WCB or WCB+BSTM were introduced to the precision model.


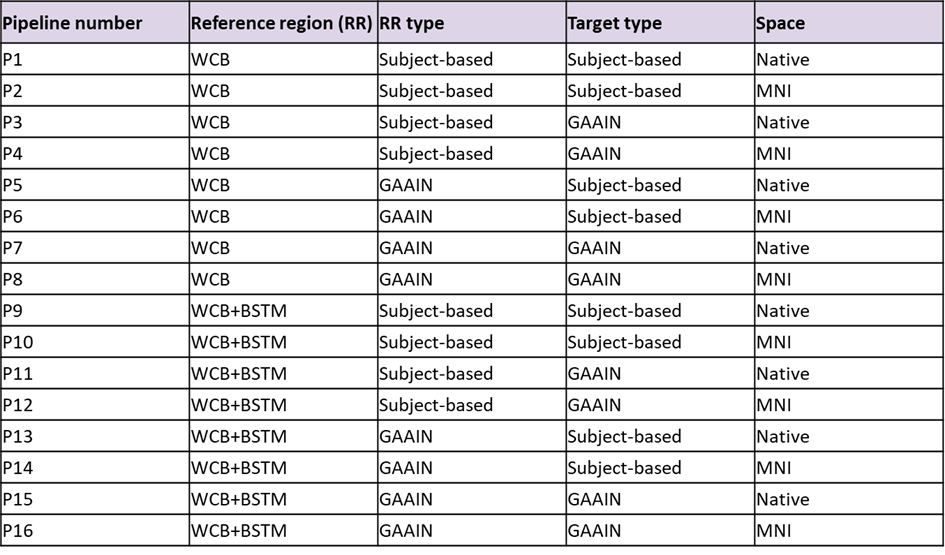


Table_s2. Combination of technical factors for each pipeline number. P8 refers to the standard GAAIN pipeline.

**Secondary ‘bias models’**

Table_s3 shows the main output of the secondary Generalized Estimation Equation (GEE) model when adding age and its interactions with other technical factors including RR selection, RR type, target type, and quantification space for amyloid-negative (A) and amyloid-positive (B) groups.


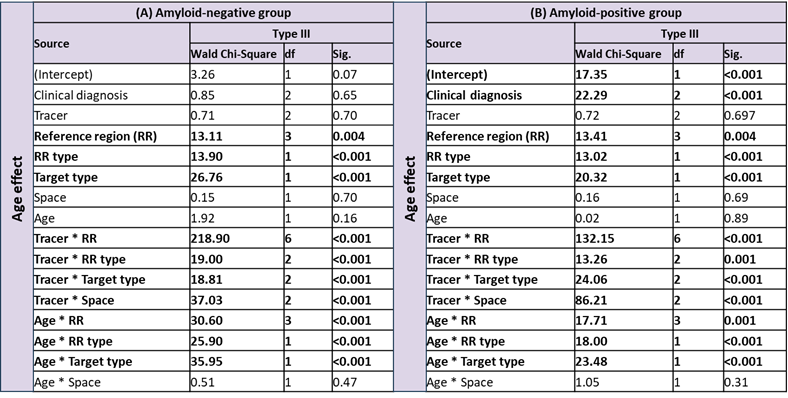


Table_s3. The main output of the base GEE model for amyloid negative and positive groups respectively (A & B). [*] refers to the interaction between factors.

**Brain Atrophy**

Table_s4 shows the output of the secondary GEE model for both amyloid negative and positive groups. This model includes the main technical factors, and brain atrophy and its interactions with technical factors. By introducing normalized gray matter volume, which is an indicator of global atrophy, quantification space is not significant anymore for both amyloid negative and positive groups.


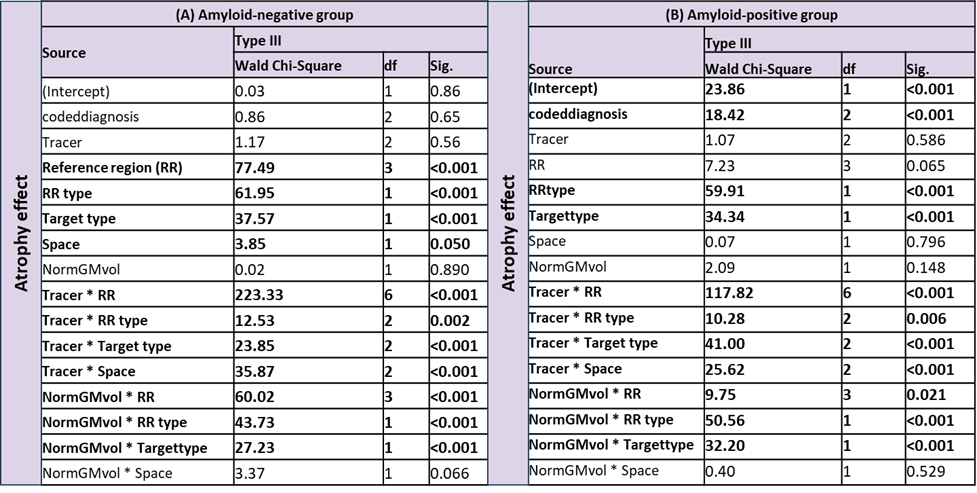


Table_s4. The main output of the base GEE model after including normalized gray matter volume and its interactions with technical factors for amyloid negative and positive respectively (A & B). [*] refers to the interaction between factors. NormGMvol (Brain atrophy): Normalized gray matter volume to total intracranial volume

Figure_s5 shows a visual example of T1-weighted MRIs of two participants with the minimum level of atrophy (subject1) and a participant with a higher extent of atrophy (Subject2) fused with corresponding GAAIN predefined cortical target (A’ and C’) and subject-based cortical target (B’ and D’) ROIs. It shows that in the presence of atrophy, the GAAIN predefined cortical target ROI covers the areas with atrophy, resulting in underestimating PET signals and subsequently Centiloid values. On the contrary, in the case of subject 2, where there is a minimal level of atrophy, both cortical target ROIs cover similar brain regions, resulting in comparable CL values (∆CL~4).


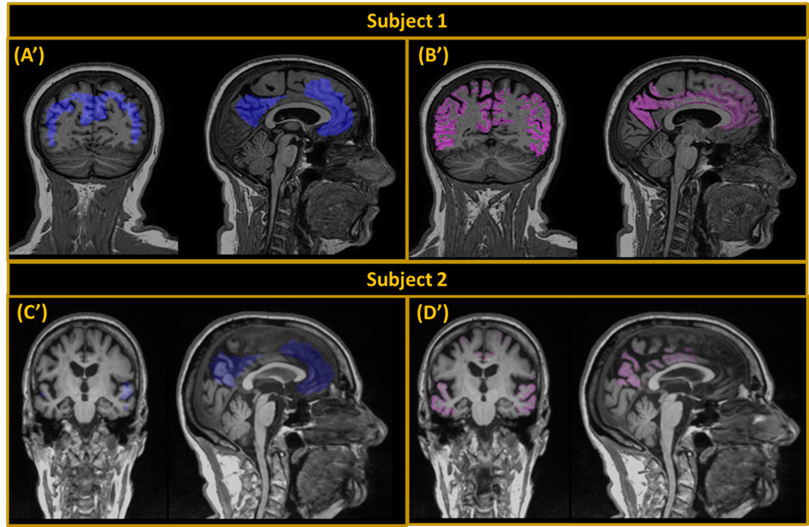


Figure_s5. Panel (A’) and (B’) show coronal and sagittal views of the T1-weighted MRI image of a 54-year-old patient with minimum atrophy, with a CL=90 and diagnosed with dementia fused with GAAIN cortical ROI and subject-based ROI respectively. Panel (C’) and (D’) show a clinical example of coronal and sagittal views of T1-weighted MRI fused with GAAIN cortical target ROI and subject-based cortical target ROI respectively. The patient was 80 years old and diagnosed with dementia with the presence of atrophy and a CL=140.

**Harmonization effect**

The harmonization effect was evaluated using a secondary GEE model. This model includes the main technical factors, harmonization status, and its interactions with technical factors. Table_s5 shows the output of the secondary GEE model for both amyloid negative and positive groups.


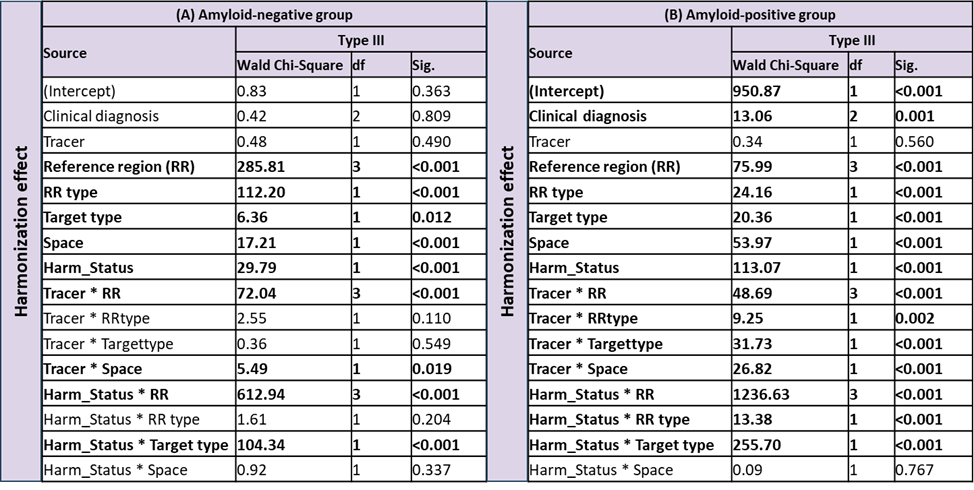


Table_s5. The main output of the GEE model after including the harmonization status and its interactions with other technical factors for amyloid-negative (A) and amyloid-positive (B) groups. This analysis includes only DPMS data where both original and harmonized data were available. [*] refers to the interaction between factors. Harm_status: Harmonization status (original vs. harmonized).

**Precision model Results**

Figure_s6 shows between-pipeline differences (marginal means and 95%CI) for both amyloid-negative and amyloid-positive groups.


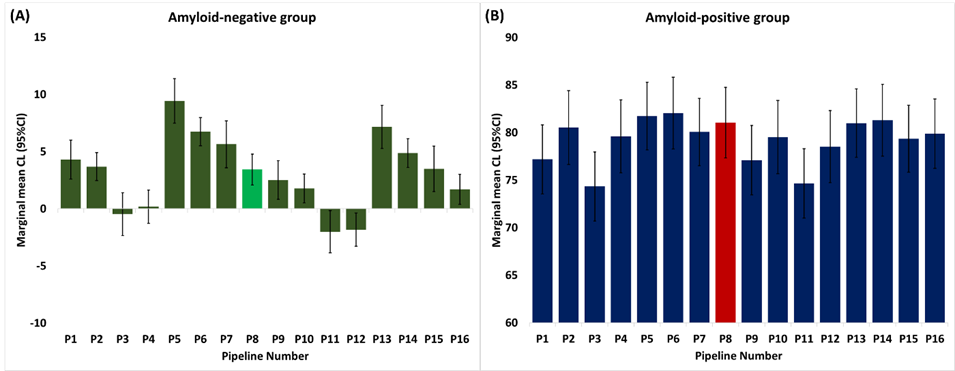


Figure_s6. Bar charts showing marginal means and corresponding 95%CI for each pipeline in (A) amyloid-negative and (B) amyloid-positive groups. Light green and red bars indicate the GAAIN standard pipeline for amyloid-negative and amyloid-positive groups respectively.


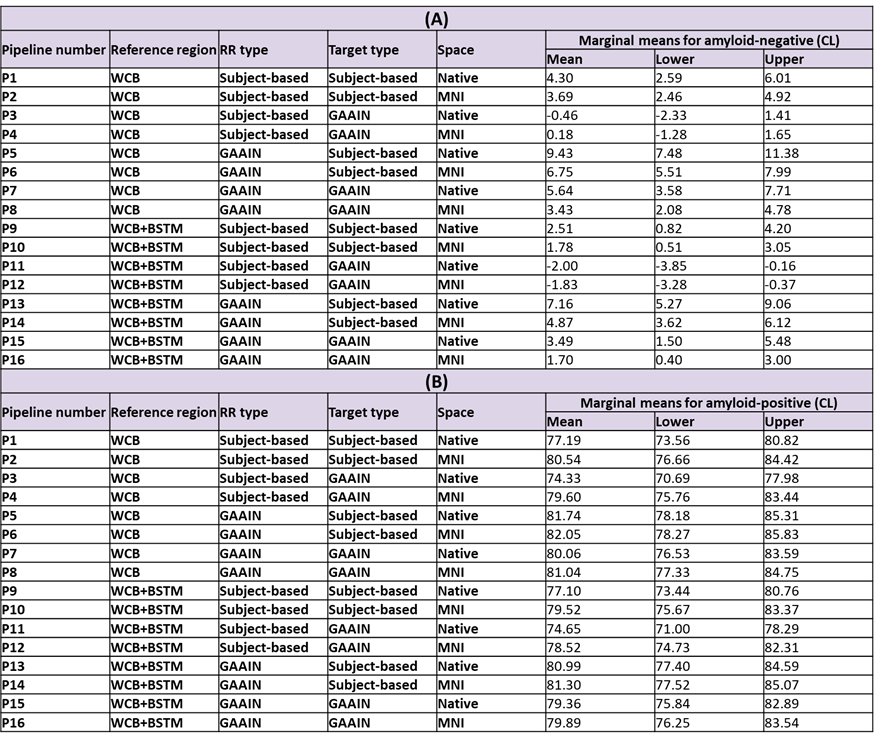


Table_s6. Marginal means for each pipeline and corresponding lower and upper 95% Wald confidence intervals for amyloid-negative (A) and amyloid-positive (B) groups.


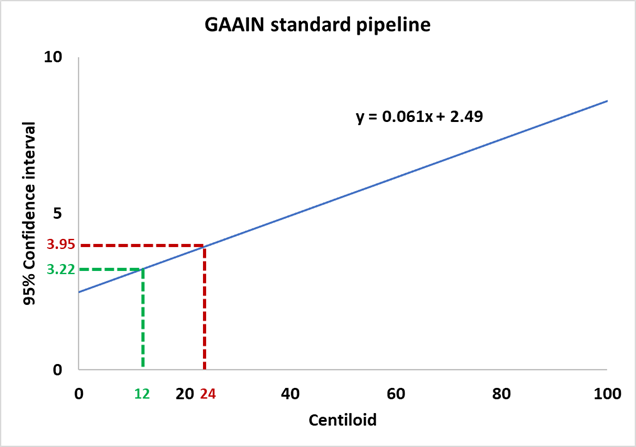


Figure_s7. Within pipeline differences for the GAAIN standard pipeline and corresponding 95% confidence intervals. The green dashed line corresponds to 95%CI while using 12 CL for defining the absence of amyloid plaques and the red dashed line corresponds to the 95% CI for defining the visual read positivity of amyloid PET scans.

**Impact of age on the CL values in each reference region**

Figure_s8 shows the discrepancies in the Centiloid values using CGM (A & B), WCB+BSTM (C & D), and Pons (E & F) vs. WCB as RR for all 3 tracers. Inverse associations between age and differences in CL when using WCB vs. Pons were observed for all 3 tracers irrespective of amyloid status. However, in the negative group, spearman’s correlation was statistically significant only for FMM, and for the amyloid-positive group, only FBB showed a significant correlation between age and ∆CL(Pons-WCB) (Table_s7, panel (A) and (B)). By comparing the correlation coefficients between different tracers and RRs, FBB is the only tracer that showed statistically significant differences with FMM and FBP for the amyloid-positive group, when using WCB+BSTM or Pons as RR (Table_s7, panel (C) and (D)). It means that part of the between tracer differences for FBB could be explained by the age effect. Nevertheless, it is important to note that the interaction between the Reference Region and Tracer showed a similar Chi-Squared value, suggesting that the between-tracer differences in CL values were not completely driven by age.

| 1. Amyloid-negative group   Spearman’s correlation (95% CI; p-values) | | | |
| --- | --- | --- | --- |
| Tracer | **CGM** | **WCB+BSTM** | **Pons** |
| FMM | **0.19 (0 to 0.37; p=0.05)** | **-0.36 (-0.52 to -0.18; p<0.001)** | **-0.33 (-0.50 to -0.15; p<0.001)** |
| FBB | 0.11 (-0.20 to 0.40; p=0.40) | -0.25 (-0.51 to 0.06; p=0.10) | -0.22 (-0.49 to 0.08; p=0.14) |
| FBP | -0.02 (-0.23 to 0.19; p=0.85) | -0.10 (-0.31 to 0.12; p=0.35) | -0.09 (-0.29 to 0.13; p=0.42) |
| 1. Amyloid-positive group   Spearman’s correlation (95% CI; p-values) | | | |
| Tracer | **CGM** | **WCB+BSTM** | **Pons** |
| FMM | 0.11 (-0.09 to 0.31; p=0.26) | **-0.20 (-0.39 to 0.00; p=0.04)** | -0.18 (-0.37 to 0.02; p=0.06) |
| FBB | **0.37 (0.16 to 0.55; p<0.001)** | **-0.52 (-0.67 to -0.33; p<0.001)** | **-0.51 (-0.66 to -0.31; p<0.001)** |
| FBP | 0.06 (-0.25 to 0.13; p=0.51) | 0.001 (-0.19 to 0.19; p=0.99) | 0.02 (-0.17 to 0.21; p=0.83) |
| 1. Amyloid-negative group   Comparing Spearman’s correlations between tracers | | | |
| Tracers | **Z_obs_ (CGM)** | **Z_obs_ (WCB+BSTM)** | **Z_obs_ (Pons)** |
| FMM vs FBB | 0.44 | -0.69 | -0.66 |
| FMM vs FBP | 1.47 | -1.91 | -1.78 |
| FBB vs FBP | 0.71 | -0.80 | -0.74 |
| 1. Amyloid-positive group   Comparing Spearman’s correlations between tracers | | | |
| Tracers | **Z_obs_ (CGM)** | **Z_obs_ (WCB+BSTM)** | **Z_obs_ (Pons)** |
| FMM vs FBB | -1.80 | **2.40** | **2.40** |
| FMM vs FBP | 1.27 | -1.48 | -1.49 |
| FBB vs FBP | **3.02** | **-3.83** | **-3.84** |

Table_s7. Spearman correlation coefficients and 95% confidence interval (95%CI) between age and differences in the CL when using WCB vs. CGM, WCB+BSTM, and Pons as RR for both amyloid negative (A) and positive (B) groups. Values highlighted in bold correspond to statistically significant correlations. Panel (C) and (D) Compare the correlation coefficients between age and ∆CL for each RR among 3 different tracers for the amyloid-negative and amyloid-positive groups respectively.

*
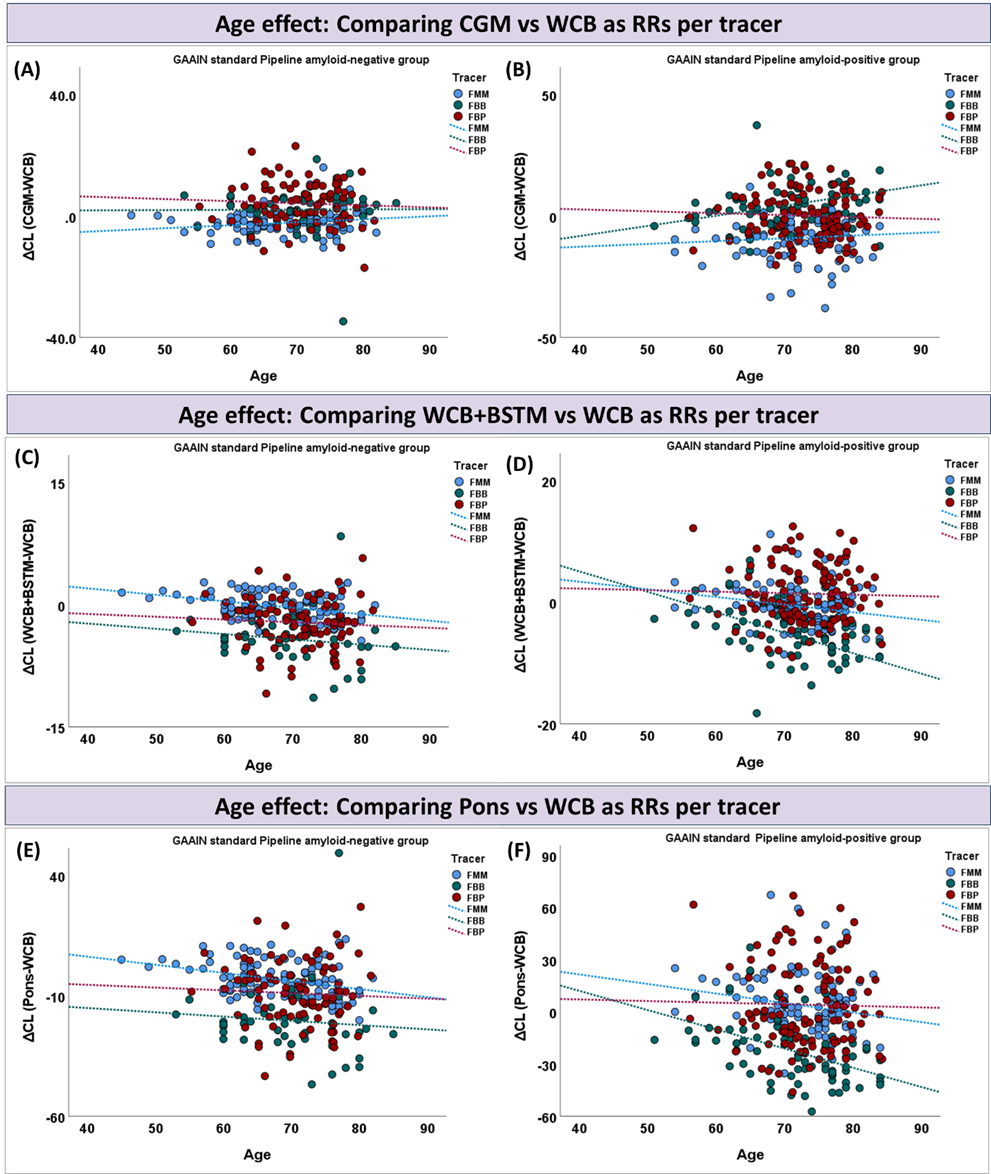
*

Figure_s8. Scatter plots showing the differences in CL when using (A & B) WCB vs CGM and (C & D) WCB vs WCB+BSTM, and (E & F) WCB vs Pons as RR for all 3 tracers with respect to age. The left panels correspond to amyloid-negative and the right panels correspond to amyloid-positive scans

**Centiloid pipeline designs**

Using the standard SPM Centiloid pipeline in the GAAIN Centiloid project website (https://www.gaain.org/centiloid-project) as the reference, we created 32 different variations, all based on SPM12, (Wellcome Centre for Human Neuroimaging, London, UK), with all possible combinations of choices of the following pipeline design options: Reference region (Whole Cerebellum, Cerebellum Gray Matter, Pons and Whole Cerebellum plus Brainstem), reference region type (GAAIN and ‘subject-based’), cortical target definition (GAAIN and ‘subject-based’), and quantification space (MNI and subject space).

**Reference region and reference region type**

Four reference regions (RR) including whole cerebellum (WCB), cerebellum gray matter (CGM), pons, and whole cerebellum plus brainstem (WCB+BSTM) were used for the quantitative normalization of PET images. Two types of RR including “GAAIN RR”, or “Subject-based RR” were used. “GAAIN RR” refers to predefined RR region of interest (ROIs) available on the GAAIN website.

"Subject-based” reference regions were delineated based on probability maps of gray matter, white matter, and cerebrospinal fluid derived from T1-weighted MRI segmentations in SPM12. To elaborate, a binary mask for gray matter was generated, including voxels with higher probabilities than those in the white matter and cerebrospinal fluid masks. Using a similar approach, a binary white matter mask was established, and the subject-based RR was derived by overlaying the Hammer atlas onto these binary gray and white matter masks (Figure_s9).


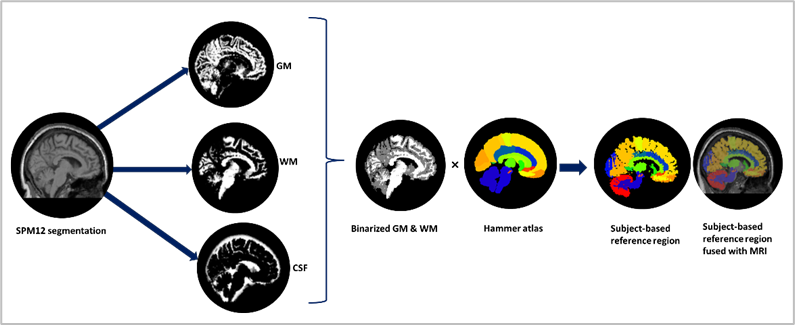


Figure_s9. Schematic of extracting subject-base reference region

**Cortical target type**

The amyloid burden was estimated using “GAAIN Crtx” or “subject-based” cortical target ROIs. “GAAIN Crtx” refers to the standard cortical mask defined on the GAAIN website, and the “Subject-based” cortical target is a composite ROI created using the AAL atlas, consisting of brain regions representing high amyloid load in AD (Table_s8).

**AAL-based composite ROI**

AAL-based composite ROIs fused with a T1-weighted MRI template is shown in Figure_s10. This composite is the input for the Centiloid pipeline before applying any preprocessing. A “subject-based” cortical target was created by mapping the binary GM mask on the AAL-based composite ROIs.


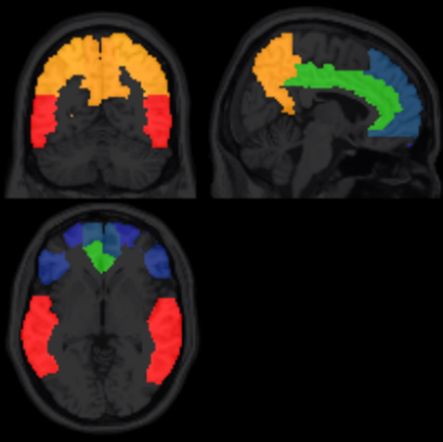


Table_s8. Brain regions and their corresponding indexes included

AAL-based composite ROI.


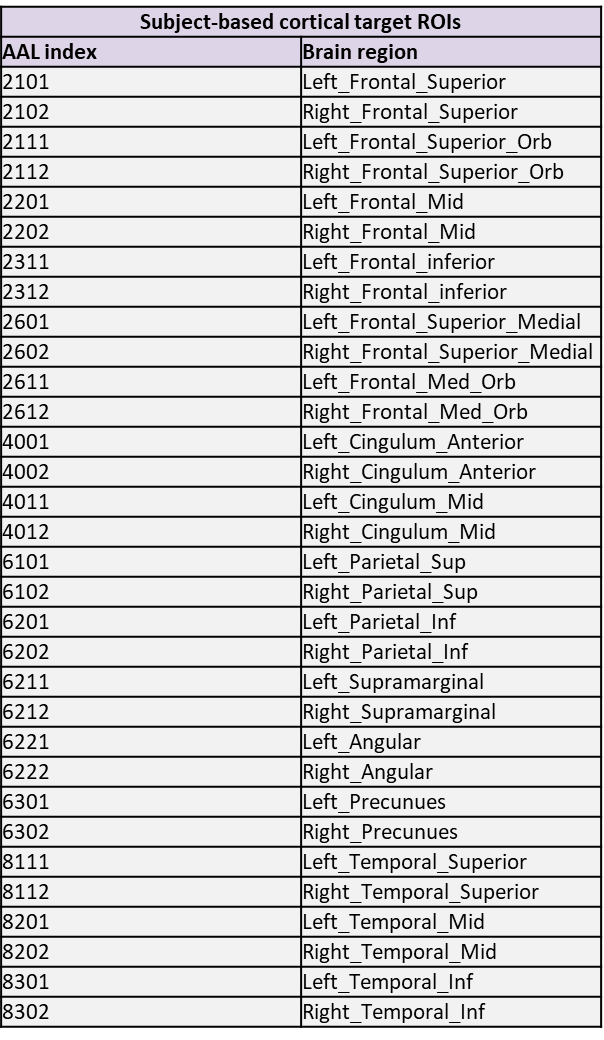


Figure_s10. AAL-based composite ROI fused with the T1-weighted MRI template prior to applying any preprocessing.

**Subject-based cortical target**

A Schematic of the steps used for extracting the subject-based cortical target is shown in Figure_s11. In brief, the T1-weighted MRI image is segmented using SPM12 segmentation function, and as output GM, WM, and CSF tissue probability maps, as well as deformation fields are provided. In the next step, a binary gray matter mask will be created that includes voxels in the GM tissue probability map (TPM) with higher values than WM and CSF TPMs. An AAL-based ROI composite will be transformed into native space using the inverse deformation field. Finally, a subject-based cortical target will be created by multiplying the binarized GM mask with the transformed AAL-composite ROI.


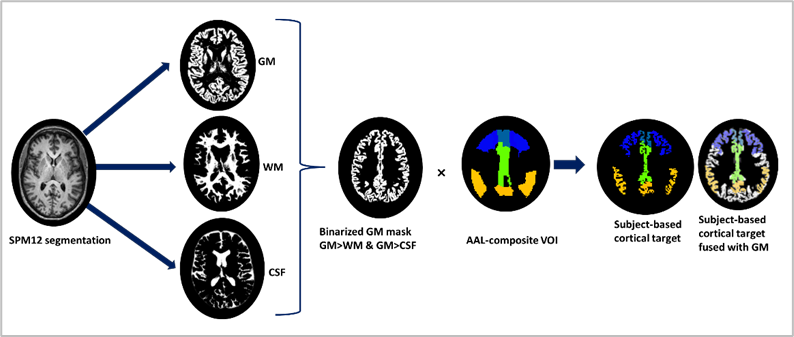


Figure_s11. Schematic of the preprocessing steps for defining subject-based cortical target using AAL-based composite ROI.

**Quantification space**

Images were quantified in either the subject space or Montreal Neurological Institute (MNI) space. Subject space was defined using T1-weighted MRI and in this space. MNI space images were created by spatially normalizing MRIs using SPM12 and applying the estimated warping maps to the PET images that were previously coregistered to the MRI scan.

**Quality Control**

Quality control of preprocessing steps and outputs of the pipelines was done visually for all images prior to calculating Centiloid values.

**Calculating conversion equation**

All the Centiloid conversion equations for our in-house pipeline were extracted using the reference dataset available on the GAAIN website. Here is a brief explanation of stepwise calibration:

**First-level calibration for 11C-PiB:**

For the first level calibration, we downloaded the ^11^C-PiB reference data set. It includes PET and T1-weighted MRI scans for 45 AD patients and 34 young control groups. Visual quality control was done on both MRI and PET scans. MRI scans were segmented using the SPM12 segmentation function to create GM (c1), WM (c2), and CSF (c3) segments as well as forward, and inverse deformation fields (y, and iy). PET images were coregistered with corresponding T1-weighted MRI. In the next step, PET scans were wrapped and moved to the MNI space using the deformation field and the “normalize” function of the SPM12. All wrapped PET images were visually checked to ensure the correct performance and transformation of the PET scans. In the next step, PET voxel intensities were divided into the mean activity in the reference region, and SUVr maps were created. Finally, cortical uptake SUVr was calculated as the mean activity concentration of the GAAIN cortical target mask into the mean activity concentration of the reference region. The first-level calibration anchor points for the in-house pipeline were calculated using the following equation:

**Centiloid=((SUVr-Mean(SUVr_YC_)))/((Mean(SUVr_AD_)-Mean(SUVr_YC_)))×100**

**Second-level calibration:**

Second-level calibration was performed for 3 ^18^F-tracers. Here, steps are defined for the ^18^F-Flutemetamol (FMM), and it is generalizable to all other tracers. In the first step, head-to-head ^11^C-PiB and ^18^F-Flutemetamol PET scans, and corresponding T1-weighted MRI were downloaded from the GAAIN website. The datasets consist of 50-70 min ^11^C-PIB and 90-110 min ^18^F-flutemetamol scans from 17 AD Patients and 23 young healthy controls and the corresponding MR data. In the first step, a visual QC was done on all images to correct for any possible mis-orientation. In the next step, all PET images (both ^11^C-PiB and ^18^F-flutemetamol scans) were wrapped and moved to the MNI space following the steps described in the first-level analysis. SUVr values were calculated for each head-to-head dataset, and using linear regression, the equations for transforming ^18^F-flutemetamol SUVr to ^11^C-PiB equivalent SUVr was calculated (FMM_to_PiB_SUVr). Then, using the anchor points calculated from the first-level calibration, FMM_to_PiB_SUVr converted to Centiloid (CLFMM). Finally, using linear regression between CL^FMM^ and SUVr^FMM^, Centiloid transformation equations were calculated for each reference region.

**CL transformation equations**

Centiloid calibration equations for ^18^F-Flutemetamol (FMM), ^18^F-Florbetaben (FBB), and ^18^F-Florbetapir (FBP) are shown in Table_s9, Table_s10, and Table_s11 respectively.


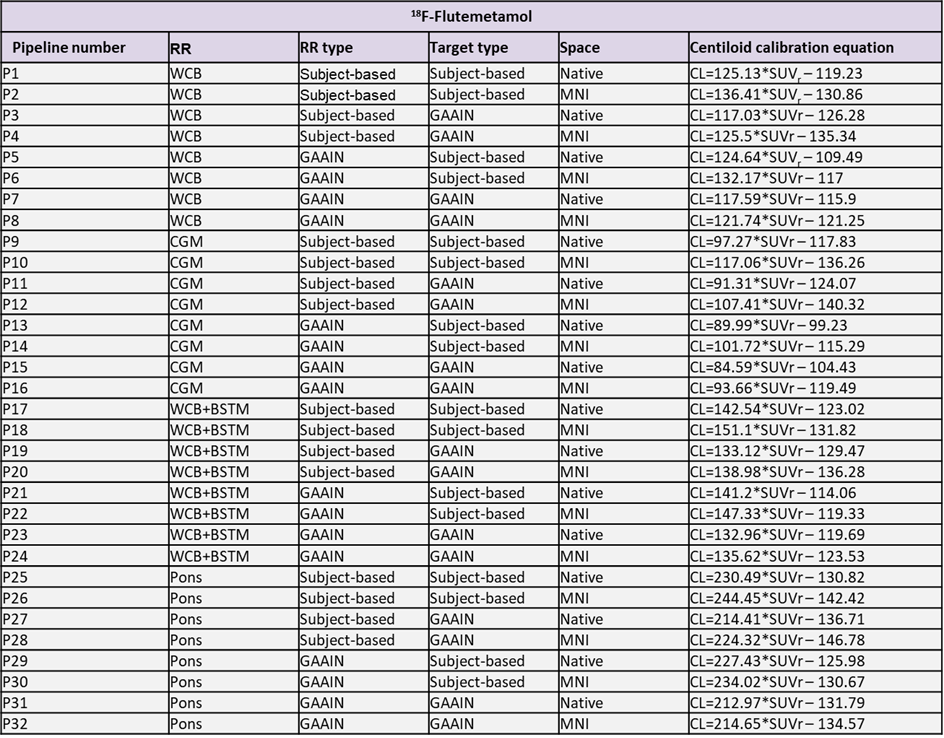


Table_s9. Centiloid equations for 32 different pipelines using the ^18^F-Flutemetamol amyloid PET tracer.


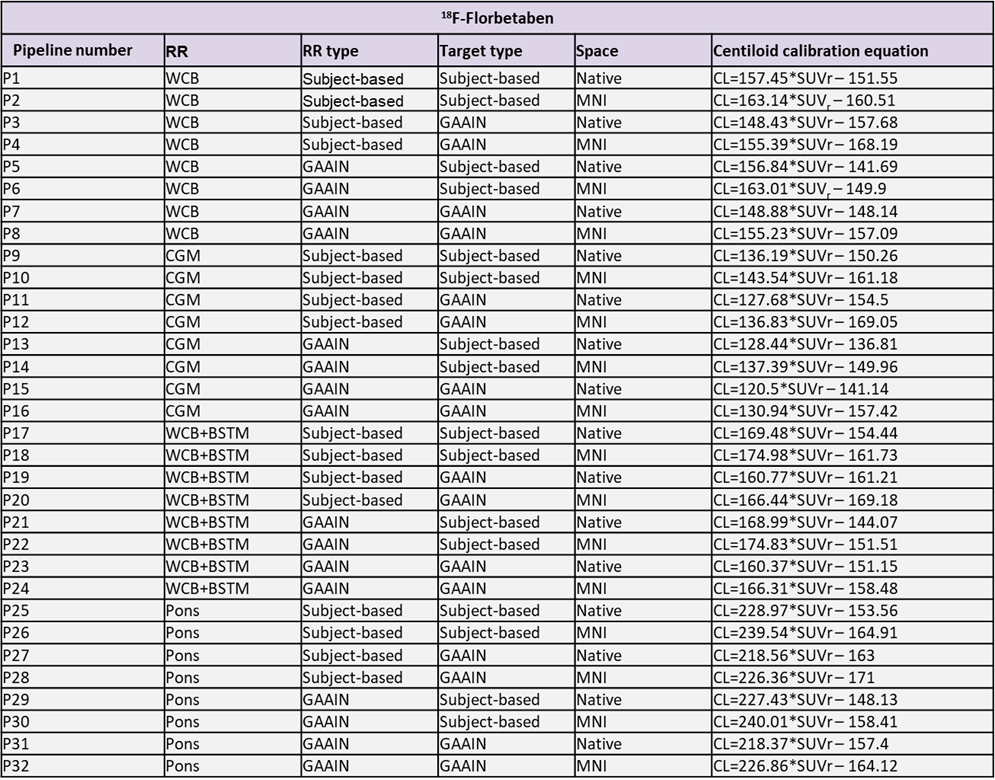


Table_s10. Centiloid equations for 32 different pipelines using the ^18^F-Florbetaben amyloid PET tracer.


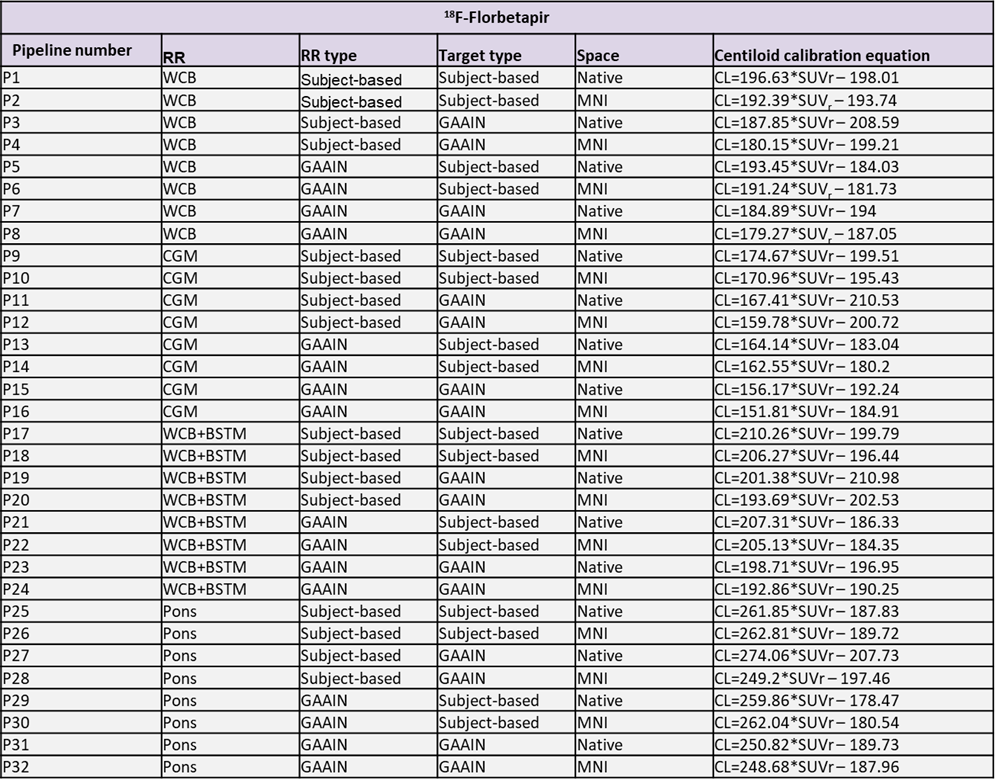


Table_s11 .Centiloid equations for 32 different pipelines using the ^18^F-Florbetapir amyloid PET tracer

**Quality control of the CL pipelines**

Quality control of different pipelines using recommendations in the Klunk et al paper ^2^:

R^2^≥ 0.98

-2 ≤ Intercepts ≤ 2

Quality control criteria of the first level calibration of Centiloid is shown in Table_s12. The correlations between the extracted SUVr from our in-house pipeline and GAAIN reference quantifications were calculated (Table_s12). ^11^C-PiB GAAIN reference quantification can be found on the GAAIN website. As shown in Table_s12, both R^2^ and intercepts are falling into the acceptance level, reassuring to define our in-house pipeline anchor points for first-level calibration. Also, Table_s13, Table_s14, and Table_s15 show the quality control criteria for the second-level analysis of FBB, FMM, and FBP respectively.


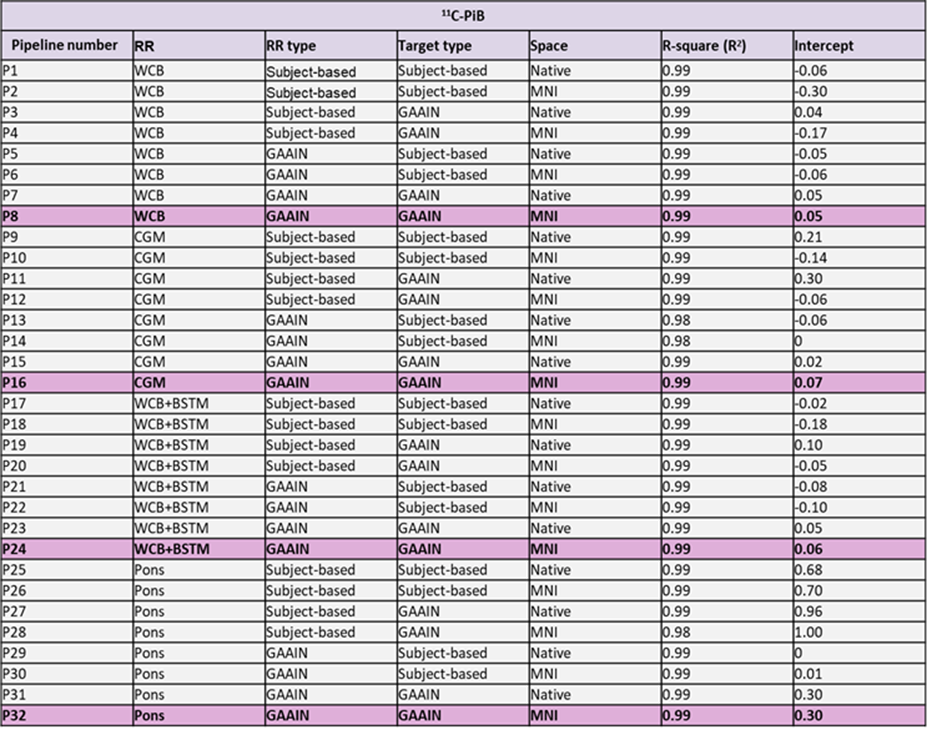


Table_s12. Correlation coefficient (R^2^) and intercept of the fitting line comparing Centiloid values extracted from the in-house pipeline vs. the reference GAAIN pipeline for first-level calibration using ^11^C-PiB. Rows highlighted in pink correspond to the predefined GAAIN RR and GAAIN cortical target ROIs and quantification in MNI space.


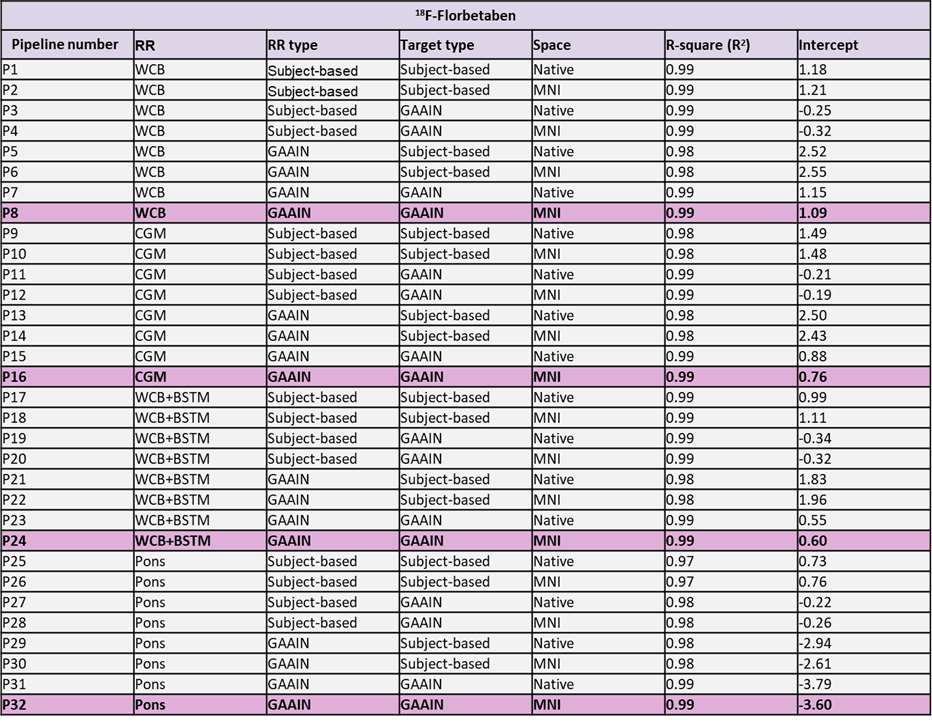


Table_s13. Correlation coefficient (R^2^) and intercept of the fitting line comparing Centiloid values extracted from the in-house pipeline vs. the reference GAAIN pipeline for first-level calibration using ^18^F-Florbetaben. Rows highlighted in pink correspond to the predefined GAAIN RR and GAAIN cortical target ROIs and quantification in MNI space.


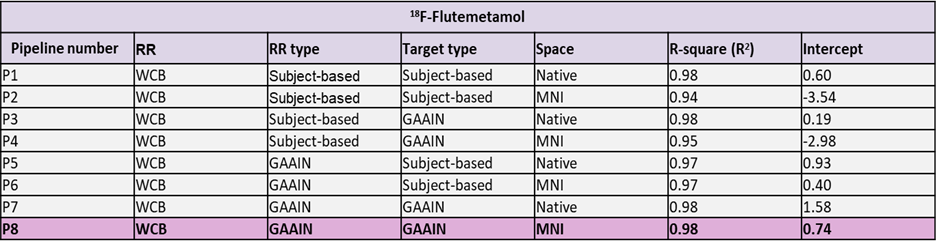


Table_s14. Correlation coefficient (R2) and intercept of the fitting line comparing Centiloid values extracted from the in-house pipeline vs. the reference GAAIN pipeline for first-level calibration using 18F-Flutemetamol. Rows highlighted in pink correspond to the predefined GAAIN RR and GAAIN cortical target ROIs and quantification in MNI space.


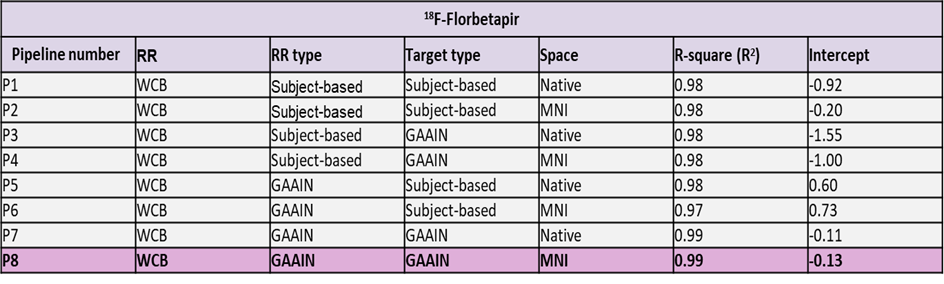


Table_s15. Correlation coefficient (R^2^) and intercept of the fitting line comparing Centiloid values extracted from the in-house pipeline vs. the reference GAAIN pipeline for first-level calibration using ^18^F-Florbetapir. Rows highlighted in pink correspond to the predefined GAAIN RR and GAAIN cortical target ROIs and quantification in MNI space.

**Defining amyloid positivity for stratifying analysis**

Analyses were stratified by amyloid positivity and considered positive if the CL value was above 24 as calculated with the standard CL pipeline ^3^. The motivation for the stratification is twofold. On the one hand, the main outcomes of this study are the marginal mean differences and 95% Confidence Intervals (95%CI), in CL units, between levels of the studied factors. Therefore, the relevance of a given CL difference is expected to be much higher in the low CL range than for higher CL values. On the other hand, this ensured the residuals of the linear models to be normally distributed, thus meeting the condition for them to provide unbiased estimates.

**Global brain atrophy**

Global atrophy was defined as total GM volume normalized to Total Intracranial Volume (TIV). For each subject, a binary mask was created by including voxels with higher probabilities of being GM than being WM or CSF. Using the same method, WM and CSF binary masks were created and TIV was calculated by summing the non-zero voxels in GM, WM, and CSF binary masks. Global atrophy is calculated by dividing the number of non-zero voxels in binary GM to TIV.

**References:**

1. Lilja J, Leuzy A, Chiotis K, Savitcheva I, Sörensen J, Nordberg A. Spatial normalization of 18F-flutemetamol PET images using an adaptive principal-component template. *Journal of Nuclear Medicine*. 2019;60(2):285-291.

2. Klunk WE, Koeppe RA, Price JC, et al. The Centiloid Project: standardizing quantitative amyloid plaque estimation by PET. *Alzheimer's & dementia*. 2015;11(1):1-15. e4.
