# Supplementary material for: Stress testing the Centiloid: Precision and variability of PET quantification of amyloid pathology
Source: Alzheimers Dement. 2024 Jul 4;20(8):5102–13. doi: 10.1002/alz.13883 (PMC11350134; doi:10.1002/alz.13883)
Supplement: Supplementary file 2 — Supporting Information [file ALZ-20-5102-s001.docx]

AMYPAD & ADNI Collaborators

| *Group Name(s): AMYPAD consortium | | | | | | | |
| --- | --- | --- | --- | --- | --- | --- | --- |
| *First Name and  Middle Initial(s) | ***Last Name** | ***Suffix**  **(eg, Jr, III)** | **Academic**  **Degrees** | **Institution** | **Location (city,**  **state/province, country)** | **Role or Contribution,**  **eg, chair, principal**  **investigator** | Group (if more than 1  Group listed in the  byline) and/or Subgroup  (eg, Steering Committee) |
| Carla | Abdelnour |  |  |  |  |  |  |
| Nuria | Aguilera |  |  |  |  |  |  |
| Leon | Aksman |  |  |  |  |  |  |
| Emilio | Alarcón‐Martín |  |  |  |  |  |  |
| Montse | Alegret |  |  |  |  |  |  |
| Silvia | Alonso‐Lana |  |  |  |  |  |  |
| Daniele | Altomare |  |  |  |  |  |  |
| Pia | Andersen |  |  |  |  |  |  |
| Majd | Arab |  |  |  |  |  |  |
| Malin | Aspö |  |  |  |  |  |  |
| Ilona | Bader |  |  |  |  |  |  |
| Ilse | Bader |  |  |  |  |  |  |
| Nigel | Banton |  |  |  |  |  |  |
| Frederik | Barkhof |  |  |  |  |  |  |
| Rodrigo | Barnes |  |  |  |  |  |  |
| Dawn | Barrie |  |  |  |  |  |  |
| Mark | Battle |  |  |  |  |  |  |
| Ana | Belén Collado |  |  |  |  |  |  |
| Julie | Bellet |  |  |  |  |  |  |
| Johannes | Berkhof |  |  |  |  |  |  |
| Marine | Biger |  |  |  |  |  |  |
| Cindy | Birck |  |  |  |  |  |  |
| Gerard | Bischof |  |  |  |  |  |  |
| Mercè | Boada |  |  |  |  |  |  |
| Ronald | Boellaard |  |  |  |  |  |  |
| Nenad | Bogdanovic |  |  |  |  |  |  |
| Ariane | Bollack |  |  |  |  |  |  |
| Stéphanie | Bombois |  |  |  |  |  |  |
| Stefan | Borg |  |  |  |  |  |  |
| Anne | Borjesson‐Hanson |  |  |  |  |  |  |
| Valdimir | Boskov |  |  |  |  |  |  |

| *First Name and Middle Initial(s) | *Last Name | *Suffix (eg, Jr, III) | Academic Degrees | Institution | Location (city, state/province, country) | Role or Contribution, eg, chair, principal investigator | Group (if more than 1 Group listed in the  byline) and/or Subgroup  (eg, Steering Committee) |
| --- | --- | --- | --- | --- | --- | --- | --- |
| Justine | Boutantin |  |  |  |  |  |  |
| Claire | Boutoleau‐Bretonniere |  |  |  |  |  |  |
| Femke | Bouwman |  |  |  |  |  |  |
| Laetitia | Breuilh |  |  |  |  |  |  |
| Eva | Bringman |  |  |  |  |  |  |
| Baptiste | Brunel |  |  |  |  |  |  |
| Marco | Bucci |  |  |  |  |  |  |
| Chris | Buckley |  |  |  |  |  |  |
| Mar | Buendía |  |  |  |  |  |  |
| Santi | Bullich |  |  |  |  |  |  |
| Anna | Calvet |  |  |  |  |  |  |
| Laia | Cañada |  |  |  |  |  |  |
| Marta | Cañada |  |  |  |  |  |  |
| Camilla | Caprioglio |  |  |  |  |  |  |
| Jorge | Cardoso |  |  |  |  |  |  |
| Jasmine | Carlier |  |  |  |  |  |  |
| Elise | Carre |  |  |  |  |  |  |
| Isabelle | Carrie |  |  |  |  |  |  |
| Pascaline | Cassagnaud |  |  |  |  |  |  |
| Emmanuelle | Cassol |  |  |  |  |  |  |
| Miguel | Castilla‐Martí |  |  |  |  |  |  |
| Elodie | Cazalon |  |  |  |  |  |  |
| Tiphaine | Chaarriau |  |  |  |  |  |  |
| Rachel | Chaigeau |  |  |  |  |  |  |
| Taylor | Chalmers |  |  |  |  |  |  |
| Marie‐Thérèse | Clerc |  |  |  |  |  |  |
| Montserrat | Clerigue |  |  |  |  |  |  |
| Emmanuel | Cognat |  |  |  |  |  |  |
| Nina | Coll |  |  |  |  |  |  |
| Lyduine E. | Collij |  |  |  |  |  |  |
| Peter | Connely |  |  |  |  |  |  |
| Elodie | Cordier |  |  |  |  |  |  |
| Corine | Costes |  |  |  |  |  |  |

| *First Name and Middle Initial(s) | *Last Name | *Suffix  (eg, Jr, III) | Academic Degrees | Institution | Location (city, state/province, country) | Role or Contribution, eg, chair, principal  investigator | Group (if more than 1 Group listed in the  byline) and/or Subgroup  (eg, Steering Committee) |
| --- | --- | --- | --- | --- | --- | --- | --- |
| Camille | Coulange |  |  |  |  |  |  |
| Hélène | Courtemanche |  |  |  |  |  |  |
| Eric | Creisson |  |  |  |  |  |  |
| Charlotte | Crinquette |  |  |  |  |  |  |
| Rosario | Cuevas |  |  |  |  |  |  |
| Marie‐Noëlle | Cufi |  |  |  |  |  |  |
| Sophie | Dardenne |  |  |  |  |  |  |
| Maria | de Arriba |  |  |  |  |  |  |
| Casper | de Costa Luis |  |  |  |  |  |  |
| Yvonne | de Gier |  |  |  |  |  |  |
| Delphine | de Verbizier Lonjon |  |  |  |  |  |  |
| Veronique | Dekker |  |  |  |  |  |  |
| Bérengère | Dekyndt |  |  |  |  |  |  |
| Xavier | Delbeuck |  |  |  |  |  |  |
| Julien | Delrieu |  |  |  |  |  |  |
| Jean‐François | Demonet |  |  |  |  |  |  |
| Vincent | Deramecourt |  |  |  |  |  |  |
| Françoise | Desclaux |  |  |  |  |  |  |
| Carlos | Diaz |  |  |  |  |  |  |
| Susana | Diego |  |  |  |  |  |  |
| Mehdi | Djafar |  |  |  |  |  |  |
| Britta | Dölle |  |  |  |  |  |  |
| Laura | Doull |  |  |  |  |  |  |
| Laurence | Dricot |  |  |  |  |  |  |
| Alexander | Drzezga |  |  |  |  |  |  |
| Bruno | Dubois |  |  |  |  |  |  |
| Julien | Dumont |  |  |  |  |  |  |
| Jean | Dumur |  |  |  |  |  |  |
| Julien | Dumurgier |  |  |  |  |  |  |
| Martin | Dvorak |  |  |  |  |  |  |
| Mirian | Ecay |  |  |  |  |  |  |
| Paul | Edison |  |  |  |  |  |  |

| ***First Name and** | ***Last Name** | ***Suffix** | **Academic** | **Institution** | **Location (city,** | **Role or Contribution,** | **Group (if more than 1** |
| --- | --- | --- | --- | --- | --- | --- | --- |
| **Middle Initial(s)** |  | **(eg, Jr, III)** | **Degrees** |  | **state/province, country)** | **eg, chair, principal** | **Group listed in the** |
|  |  |  |  |  |  | **investigator** | **byline) and/or Subgroup** |
|  |  |  |  |  |  |  | **(eg, Steering Committee)** |
| Claus | Escher |  |  |  |  |  |  |
| Ainara | Estanga |  |  |  |  |  |  |
| Ester | Esteban |  |  |  |  |  |  |
| Guy | Fanjaud |  |  |  |  |  |  |
| Gill | Farrar |  |  |  |  |  |  |
| Karine | Fauria |  |  |  |  |  |  |
| Marta | Felez Sanchez |  |  |  |  |  |  |
| Patrick | Feukam Talla |  |  |  |  |  |  |
| Lisa | Ford |  |  |  |  |  |  |
| Giovanni B. | Frisoni |  |  |  |  |  |  |
| David | Fuster |  |  |  |  |  |  |
| Audrey | Gabelle |  |  |  |  |  |  |
| Valentina | Garibotto |  |  |  |  |  |  |
| Sinead | Gaubert |  |  |  |  |  |  |
| Cédric | Gauci |  |  |  |  |  |  |
| Christine | Geldhof |  |  |  |  |  |  |
| Jean | Georges |  |  |  |  |  |  |
| Joseph | Ghika |  |  |  |  |  |  |
| Rossella | Gismondi |  |  |  |  |  |  |
| Juan Domingo | Gispert |  |  |  |  |  |  |
| Elena | González |  |  |  |  |  |  |
| Valerie | Goovaerts |  |  |  |  |  |  |
| Denis Mariano | Goulart |  |  |  |  |  |  |
| Caroline | Grasselli |  |  |  |  |  |  |
| Oriol | Grau‐Rivera |  |  |  |  |  |  |
| Katherine | Gray |  |  |  |  |  |  |
| Martin | Greensmith |  |  |  |  |  |  |
| Laure | Grozn |  |  |  |  |  |  |
| Céline | Guillemaud |  |  |  |  |  |  |
| Fiona | Gunn |  |  |  |  |  |  |
| Prasad | Guntur Ramkumar |  |  |  |  |  |  |
| Göran | Hagman |  |  |  |  |  |  |
| Bernard | Hanseeuw |  |  |  |  |  |  |

| ***First Name and**  **Middle Initial(s)** | ***Last Name** | ***Suffix**  **(eg, Jr, III)** | **Academic**  **Degrees** | **Institution** | **Location (city,**  **state/province, country)** | **Role or Contribution,**  **eg, chair, principal**  **investigator** | **Group (if more than 1**  **Group listed in the**  **byline) and/or Subgroup**  **(eg, Steering Committee)** |
| --- | --- | --- | --- | --- | --- | --- | --- |
| Fiona | Heeman |  |  |  |  |  |  |
| Janine | Hendriks |  |  |  |  |  |  |
| Jakob | Himmelmann |  |  |  |  |  |  |
| Anne | Hitzel |  |  |  |  |  |  |
| Florent | Hives |  |  |  |  |  |  |
| Merle | Hoenig |  |  |  |  |  |  |
| Claire | Hourrègue |  |  |  |  |  |  |
| Justine | Hudson |  |  |  |  |  |  |
| Jordi | Huguet |  |  |  |  |  |  |
| Marta | Ibarria |  |  |  |  |  |  |
| Ifrah | Iidow |  |  |  |  |  |  |
| Sandrine | Indart |  |  |  |  |  |  |
| Silvia | Ingala |  |  |  |  |  |  |
| Adrian | Ivanoiu |  |  |  |  |  |  |
| Charlotte | Jacquemont |  |  |  |  |  |  |
| Vesna | Jelic |  |  |  |  |  |  |
| Frank | Jessen |  |  |  |  |  |  |
| Jieqing | Jiao |  |  |  |  |  |  |
| Sara | Jofresa |  |  |  |  |  |  |
| Cathrine | Jonsson |  |  |  |  |  |  |
| Dzmitry | Kaliukhovich |  |  |  |  |  |  |
| Silke | Kern |  |  |  |  |  |  |
| Miia | Kivipelto |  |  |  |  |  |  |
| Iva | Knezevic |  |  |  |  |  |  |
| Grégory | Kuchcinski |  |  |  |  |  |  |
| Manon | Laforce |  |  |  |  |  |  |
| Asunción | Lafuente |  |  |  |  |  |  |
| Françoise | Lala |  |  |  |  |  |  |
| Adriaan | Lammertsma |  |  |  |  |  |  |
| Michelle | Lax |  |  |  |  |  |  |
| Thibaud | Lebouvier |  |  |  |  |  |  |
| Ho‐Yun | Lee |  |  |  |  |  |  |
| Lean | Lee |  |  |  |  |  |  |
| Annebet | Leeuwis |  |  |  |  |  |  |

| ***First Name and** | ***Last Name** | ***Suffix** | **Academic** | **Institution** | **Location (city,** | **Role or Contribution,** | **Group (if more than 1** |
| --- | --- | --- | --- | --- | --- | --- | --- |
| **Middle Initial(s)** |  | **(eg, Jr, III)** | **Degrees** |  | **state/province, country)** | **eg, chair, principal** | **Group listed in the** |
|  |  |  |  |  |  | **investigator** | **byline) and/or Subgroup** |
|  |  |  |  |  |  |  | **(eg, Steering Committee)** |
| Amandine | Lefort |  |  |  |  |  |  |
| Jean‐François | Legrand |  |  |  |  |  |  |
| Mélanie | Leroy |  |  |  |  |  |  |
| Constance | Lesoil Markowski |  |  |  |  |  |  |
| Marcel | Levy |  |  |  |  |  |  |
| Renaud | Lhommel |  |  |  |  |  |  |
| Renaud | Lopes |  |  |  |  |  |  |
| Isadora | Lopes Alves |  |  |  |  |  |  |
| Luigi | Lorenzini |  |  |  |  |  |  |
| Adrien | Lorette |  |  |  |  |  |  |
| Emma | Luckett |  |  |  |  |  |  |
| Marie | Lundin |  |  |  |  |  |  |
| Marie‐Anne | Mackowiak |  |  |  |  |  |  |
| Vincent | Malotaux |  |  |  |  |  |  |
| Richard | Manber |  |  |  |  |  |  |
| Nikolay | Manyakov |  |  |  |  |  |  |
| Pawel | Markiewicz |  |  |  |  |  |  |
| Paula | Marne |  |  |  |  |  |  |
| Marta | Marquié |  |  |  |  |  |  |
| Elvira | Martín |  |  |  |  |  |  |
| Joan | Martínez |  |  |  |  |  |  |
| Pablo | Martinez Lage |  |  |  |  |  |  |
| Sophie E. | Mastenbroek |  |  |  |  |  |  |
| Aurélien | Maureille |  |  |  |  |  |  |
| Karen | Meersmans |  |  |  |  |  |  |
| Anja | Mett |  |  |  |  |  |  |
| Joseph | Milne |  |  |  |  |  |  |
| Carolina | Minguillón |  |  |  |  |  |  |
| Marc | Modat |  |  |  |  |  |  |
| José Luis | Molinuevo |  |  |  |  |  |  |
| Laura | Montrreal |  |  |  |  |  |  |
| Christian | Moro |  |  |  |  |  |  |
| Theresa | Müller |  |  |  |  |  |  |

| ***First Name and** | ***Last Name** | ***Suffix** | **Academic** | **Institution** | **Location (city,** | **Role or Contribution,** | **Group (if more than 1** |
| --- | --- | --- | --- | --- | --- | --- | --- |
| **Middle Initial(s)** |  | **(eg, Jr, III)** | **Degrees** |  | **state/province, country)** | **eg, chair, principal** | **Group listed in the** |
|  |  |  |  |  |  | **investigator** | **byline) and/or Subgroup** |
|  |  |  |  |  |  |  | **(eg, Steering Committee)** |
| Graciela | Muniz |  |  |  |  |  |  |
| Henk Jan | Mutsarts |  |  |  |  |  |  |
| Ted | Nilsson |  |  |  |  |  |  |
| Aida | Ninerola |  |  |  |  |  |  |
| Agneta | Nordberg |  |  |  |  |  |  |
| Wilse | Novaes |  |  |  |  |  |  |
| Joao | Nuno Carmelo Pires Silva |  |  |  |  |  |  |
| Greg | Operto |  |  |  |  |  |  |
| Adela | Orellana |  |  |  |  |  |  |
| Pierre‐Jean | Ousset |  |  |  |  |  |  |
| Olivier | Outteryck |  |  |  |  |  |  |
| Amandine | Pallardy |  |  |  |  |  |  |
| Alessandro | Palombit |  |  |  |  |  |  |
| Ana | Pancho |  |  |  |  |  |  |
| Martin | Pappon |  |  |  |  |  |  |
| Claire | Paquet |  |  |  |  |  |  |
| Jérémie | Pariente |  |  |  |  |  |  |
| Florence | Pasquier |  |  |  |  |  |  |
| Pierre | Payoux |  |  |  |  |  |  |
| Harry | Peaker |  |  |  |  |  |  |
| Esther | Pelejà |  |  |  |  |  |  |
| Delphine | Pennetier |  |  |  |  |  |  |
| Alba | Pérez‐Cordón |  |  |  |  |  |  |
| Andrés | Perissinotti |  |  |  |  |  |  |
| Matthieu Paul | Perrenoud |  |  |  |  |  |  |
| Sandrine | Petit |  |  |  |  |  |  |
| Grégory | Petyt |  |  |  |  |  |  |
| Julia | Pfeil |  |  |  |  |  |  |
| Blanche | Pirotte |  |  |  |  |  |  |
| Sandra | Pla |  |  |  |  |  |  |
| Sonia | Plaza Wuthrich |  |  |  |  |  |  |

| ***First Name and** | ***Last Name** | ***Suffix** | **Academic** | **Institution** | **Location (city,** | **Role or Contribution,** | **Group (if more than 1** |
| --- | --- | --- | --- | --- | --- | --- | --- |
| **Middle Initial(s)** |  | **(eg, Jr, III)** | **Degrees** |  | **state/province, country)** | **eg, chair, principal** | **Group listed in the** |
|  |  |  |  |  |  | **investigator** | **byline) and/or Subgroup** |
|  |  |  |  |  |  |  | **(eg, Steering Committee)** |
| Lea | Poitrine |  |  |  |  |  |  |
| Marianne | Pollet |  |  |  |  |  |  |
| Jean‐Benoit | Poncelet |  |  |  |  |  |  |
| John | Prior |  |  |  |  |  |  |
| Jean‐Pierre | Pruvo |  |  |  |  |  |  |
| Pauline | Putallaz |  |  |  |  |  |  |
| Mathieu | Queneau |  |  |  |  |  |  |
| Lisa | Quenon |  |  |  |  |  |  |
| Andreea | Rădoi |  |  |  |  |  |  |
| Marie | Rafiq |  |  |  |  |  |  |
| Fiona | Ramage |  |  |  |  |  |  |
| Maribel | Ramis |  |  |  |  |  |  |
| Michael | Reinwald |  |  |  |  |  |  |
| Gonzalo | Rios |  |  |  |  |  |  |
| Craig | Ritchie |  |  |  |  |  |  |
| Elena | Rodriguez |  |  |  |  |  |  |
| Adeline | Rollin |  |  |  |  |  |  |
| Olivier | Rouaud |  |  |  |  |  |  |
| Simona | Sacuiu |  |  |  |  |  |  |
| Laure | Saint‐Aubert |  |  |  |  |  |  |
| Arianna | Sala |  |  |  |  |  |  |
| Anne‐Sophie | Salabert |  |  |  |  |  |  |
| Jon | Saldias |  |  |  |  |  |  |
| Gemma | Salvadó |  |  |  |  |  |  |
| Angela | Sanabria |  |  |  |  |  |  |
| Lena | Sannemann |  |  |  |  |  |  |
| Nathalie | Sastre |  |  |  |  |  |  |
| Daniela | Savina |  |  |  |  |  |  |
| Irina | Savitcheva |  |  |  |  |  |  |
| Jolien | Schaeverbeke |  |  |  |  |  |  |
| Philip | Scheltens |  |  |  |  |  |  |
| Carine | Schildermans |  |  |  |  |  |  |
| Mark | Schmidt |  |  |  |  |  |  |
| Michael | Schöll |  |  |  |  |  |  |

| ***First Name and** | ***Last Name** | ***Suffix** | **Academic** | **Institution** | **Location (city,** | **Role or Contribution,** | **Group (if more than 1** |
| --- | --- | --- | --- | --- | --- | --- | --- |
| **Middle Initial(s)** |  | **(eg, Jr, III)** | **Degrees** |  | **state/province, country)** | **eg, chair, principal** | **Group listed in the** |
|  |  |  |  |  |  | **investigator** | **byline) and/or Subgroup** |
|  |  |  |  |  |  |  | **(eg, Steering Committee)** |
| Jeroen | Schuermans |  |  |  |  |  |  |
| Franck | Semah |  |  |  |  |  |  |
| Mahnaz | Shekari |  |  |  |  |  |  |
| Ingmar | Skoog |  |  |  |  |  |  |
| Oscar | Sotolongo‐Grau |  |  |  |  |  |  |
| Andrew | Stephens |  |  |  |  |  |  |
| Tiffany | Stewart |  |  |  |  |  |  |
| Jennyfer | Stutzmann |  |  |  |  |  |  |
| Murray | Tait |  |  |  |  |  |  |
| Lluis | Tárraga |  |  |  |  |  |  |
| Juan Pablo | Tartari |  |  |  |  |  |  |
| Ann‐christine | Tysen‐backstrom |  |  |  |  |  |  |
| Sergi | Valero |  |  |  |  |  |  |
| David | Vallez Garcia |  |  |  |  |  |  |
| Bart N.M. | van Berckel |  |  |  |  |  |  |
| Martijn | van Essen |  |  |  |  |  |  |
| Koen | Van Laere |  |  |  |  |  |  |
| Jeroen | van Leur |  |  |  |  |  |  |
| Ingrid S. | van Maurik |  |  |  |  |  |  |
| Rik | Vandenberghe |  |  |  |  |  |  |
| Bruno | Vellas |  |  |  |  |  |  |
| Jukka | Virolinen |  |  |  |  |  |  |
| Pieter Jelle | Visser |  |  |  |  |  |  |
| Zuzana | Walker |  |  |  |  |  |  |
| Håkan | Walles |  |  |  |  |  |  |
| Emilia | Wallin |  |  |  |  |  |  |
| Grant | Whitelaw |  |  |  |  |  |  |
| Catriona | Wimberley |  |  |  |  |  |  |
| Zarni | Win |  |  |  |  |  |  |
| Alle Meije | Wink |  |  |  |  |  |  |
| Robin | Wolz |  |  |  |  |  |  |

| ***First Name and** | ***Last Name** | ***Suffix** | **Academic** | **Institution** | **Location (city,** | **Role or Contribution,** | **Group (if more than 1** |
| --- | --- | --- | --- | --- | --- | --- | --- |
| **Middle Initial(s)** |  | **(eg, Jr, III)** | **Degrees** |  | **state/province, country)** | **eg, chair, principal** | **Group listed in the** |
|  |  |  |  |  |  | **investigator** | **byline) and/or Subgroup** |
|  |  |  |  |  |  |  | **(eg, Steering Committee)** |
| John | Woodside |  |  |  |  |  |  |
| Maqsood | Yaqub |  |  |  |  |  |  |
| Anna | Zettergren |  |  |  |  |  |  |
| Philip | Zeyen |  |  |  |  |  |  |

| ***Group Name(s): ADNI Cohort** | | | | | | | |
| --- | --- | --- | --- | --- | --- | --- | --- |
| ***First Name and** | ***Last Name** | ***Suffix** | **Academic** | **Institution** | **Location (city,** | **Role or Contribution,** | **Group (if more than 1** |
| **Middle Initial(s)** |  | **(eg, Jr, III)** | **Degrees** |  | **state/province, country)** | **eg, chair, principal** | **Group listed in the byline)** |
|  |  |  |  |  |  | **investigator** | **and/or Subgroup (eg,** |
|  |  |  |  |  |  |  | **Steering Committee)** |
| Michael | Weiner |  |  |  |  |  |  |
| Paul | Aisen |  |  |  |  |  |  |
| Ronald | Petersen |  |  |  |  |  |  |
| Clifford R. | Jack | Jr. |  |  |  |  |  |
| William | Jagust |  |  |  |  |  |  |
| John Q | Trojanowki |  |  |  |  |  |  |
| Arthur W. | Toga |  |  |  |  |  |  |
| Laurel | Beckett |  |  |  |  |  |  |
| Robert C. | Green |  |  |  |  |  |  |
| Andrew J. | Saykin |  |  |  |  |  |  |
| John | Morris |  |  |  |  |  |  |
| Leslie M. | Shaw |  |  |  |  |  |  |
| Enchi | Liu |  |  |  |  |  |  |
| Tom | Montine |  |  |  |  |  |  |
| Ronald G. | Thomas |  |  |  |  |  |  |
| Michael | Donohue |  |  |  |  |  |  |
| Sarah | Walter |  |  |  |  |  |  |
| Devon | Gessert |  |  |  |  |  |  |
| Tamie | Sather |  |  |  |  |  |  |
| Gus | Jiminez |  |  |  |  |  |  |
| Danielle | Harvey |  |  |  |  |  |  |
| Michael | Donohue |  |  |  |  |  |  |
| Matthew | Bernstein |  |  |  |  |  |  |
| Nick | Fox |  |  |  |  |  |  |
| Paul | Thompson |  |  |  |  |  |  |
| Norbert | Schuff |  |  |  |  |  |  |
| Charles | DeCArli |  |  |  |  |  |  |
| Bret | Borowski |  |  |  |  |  |  |
| Jeff | Gunter |  |  |  |  |  |  |

| ***First Name and** | ***Last Name** | ***Suffix** | **Academic** | **Institution** | **Location (city,** | **Role or Contribution,** | **Group (if more than 1** |
| --- | --- | --- | --- | --- | --- | --- | --- |
| **Middle Initial(s)** |  | **(eg, Jr, III)** | **Degrees** |  | **state/province, country)** | **eg, chair, principal** | **Group listed in the byline)** |
|  |  |  |  |  |  | **investigator** | **and/or Subgroup (eg,** |
|  |  |  |  |  |  |  | **Steering Committee)** |
| Matt | Senjem |  |  |  |  |  |  |
| Prashanthi | Vemuri |  |  |  |  |  |  |
| David | Jones |  |  |  |  |  |  |
| Kejal | Kantarci |  |  |  |  |  |  |
| Chad | Ward |  |  |  |  |  |  |
| Robert A. | Koeppe |  |  |  |  |  |  |
| Norm | Foster |  |  |  |  |  |  |
| Eric M. | Reiman |  |  |  |  |  |  |
| Kewei | Chen |  |  |  |  |  |  |
| Chet | Mathis |  |  |  |  |  |  |
| Susan | Landau |  |  |  |  |  |  |
| Nigel J. | Cairns |  |  |  |  |  |  |
| Erin | Householder |  |  |  |  |  |  |
| Lisa | Taylor Reinwald |  |  |  |  |  |  |
| Virginia | Lee |  |  |  |  |  |  |
| Magdalena | Korecka |  |  |  |  |  |  |
| Michal | Figurski |  |  |  |  |  |  |
| Karen | Crawford |  |  |  |  |  |  |
| Scott | Neu |  |  |  |  |  |  |
| Tatiana M. | Foroud |  |  |  |  |  |  |
| Steven | Potkin |  |  |  |  |  |  |
| Li | Shen |  |  |  |  |  |  |
| Faber | Kelley |  |  |  |  |  |  |
| Sungeun | Kim |  |  |  |  |  |  |
| Kwangsik | Nho |  |  |  |  |  |  |
| Zaven | Kachaturian |  |  |  |  |  |  |
| Richard | Frank |  |  |  |  |  |  |
| Peter J. | J Snyder |  |  |  |  |  |  |
| Susan | Molchan |  |  |  |  |  |  |
| Jeffrey | Kaye |  |  |  |  |  |  |

| ***First Name and Middle Initial(s)** | ***Last Name** | ***Suffix (eg, Jr, III)** | **Academic Degrees** | **Institution** | **Location (city, state/province, country)** | **Role or Contribution, eg, chair, principal investigator** | **Group (if more than 1 Group listed in the byline) and/or Subgroup (eg, Steering Committee)** |
| --- | --- | --- | --- | --- | --- | --- | --- |
| Joseph | Quinn |  |  |  |  |  |  |
| Betty | Lind |  |  |  |  |  |  |
| Raina | Carter |  |  |  |  |  |  |
| Sara | Dolen |  |  |  |  |  |  |
| Lon S. | Schneider |  |  |  |  |  |  |
| Sonia | Pawluczyk |  |  |  |  |  |  |
| Mauricio | Beccera |  |  |  |  |  |  |
| Liberty | Teodoro |  |  |  |  |  |  |
| Bryan M. | M Spann |  |  |  |  |  |  |
| James | Brewer |  |  |  |  |  |  |
| Helen | Vanderswag |  |  |  |  |  |  |
| Adam | Fleisher |  |  |  |  |  |  |
| Judith L | Heidebrink |  |  |  |  |  |  |
| Joanne L | Lord |  |  |  |  |  |  |
| Ronald | Petersen |  |  |  |  |  |  |
| Sara | Mason |  |  |  |  |  |  |
| Colleen | Albers |  |  |  |  |  |  |
| David | Knopman |  |  |  |  |  |  |
| Kris | Johnson |  |  |  |  |  |  |
| Rachelle S | Doody |  |  |  |  |  |  |
| Javier | Villanueva Meyer |  |  |  |  |  |  |
| Munir | Chowdhury |  |  |  |  |  |  |
| Susan | Rountree |  |  |  |  |  |  |
| Mimi | Dang |  |  |  |  |  |  |
| Yaakov | Stern |  |  |  |  |  |  |
| Lawrence S | Honig |  |  |  |  |  |  |
| Karen L | Bell |  |  |  |  |  |  |
| Beau | Ances |  |  |  |  |  |  |
| John C | Morris |  |  |  |  |  |  |
| Maria | Carroll |  |  |  |  |  |  |

| ***First Name and Middle Initial(s)** | ***Last Name** | ***Suffix (eg, Jr, III)** | **Academic Degrees** | **Institution** | **Location (city, state/province, country)** | **Role or Contribution, eg, chair, principal investigator** | **Group (if more than 1 Group listed in the byline) and/or Subgroup (eg, Steering Committee)** |
| --- | --- | --- | --- | --- | --- | --- | --- |
| Sue | Leon |  |  |  |  |  |  |
| Erin | Householder |  |  |  |  |  |  |
| Mark A | Mintun |  |  |  |  |  |  |
| Stacy | Schneider |  |  |  |  |  |  |
| Angela | OliverNG |  |  |  |  |  |  |
| Randall | Griffith |  |  |  |  |  |  |
| David | Clark |  |  |  |  |  |  |
| David | Geldmacher |  |  |  |  |  |  |
| John | Brockington |  |  |  |  |  |  |
| Erik | Roberson |  |  |  |  |  |  |
| Hillel | Grossman |  |  |  |  |  |  |
| Effie | Mitsis |  |  |  |  |  |  |
| Leyla | deToledo‐  Morrell |  |  |  |  |  |  |
| Raj C | Shah |  |  |  |  |  |  |
| Ranjan | Duara |  |  |  |  |  |  |
| Daniel | Varon |  |  |  |  |  |  |
| Maria T | Greig |  |  |  |  |  |  |
| Peggy | Roberts |  |  |  |  |  |  |
| Marilyn | Albert |  |  |  |  |  |  |
| Chiadi | Onyike |  |  |  |  |  |  |
| Daniel | D’Agostino | II |  |  |  |  |  |
| Stephanie | Kielb |  |  |  |  |  |  |
| James E | Galvin |  |  |  |  |  |  |
| Dana M | Pogorelec |  |  |  |  |  |  |
| Brittany | Cerbone |  |  |  |  |  |  |
| Christina A | Michel |  |  |  |  |  |  |
| Henry | Rusinek |  |  |  |  |  |  |
| Mony J | de Leon |  |  |  |  |  |  |
| Lidia | Glodzik |  |  |  |  |  |  |
| Susan | De Santi |  |  |  |  |  |  |

| ***First Name and Middle Initial(s)** | ***Last Name** | ***Suffix (eg, Jr, III)** | **Academic Degrees** | **Institution** | **Location (city, state/province, country)** | **Role or Contribution, eg, chair, principal investigator** | **Group (if more than 1 Group listed in the byline) and/or Subgroup (eg, Steering Committee)** |
| --- | --- | --- | --- | --- | --- | --- | --- |
| P | Murali  Doraiswamy |  |  |  |  |  |  |
| Jeffrey | R Petrella |  |  |  |  |  |  |
| Terence | Z Wong |  |  |  |  |  |  |
| Steven | E Arnold |  |  |  |  |  |  |
| Jason | H Karlawish |  |  |  |  |  |  |
| David | Wolk |  |  |  |  |  |  |
| Charles | D Smith |  |  |  |  |  |  |
| Greg | Jicha |  |  |  |  |  |  |
| Peter | Hardy |  |  |  |  |  |  |
| Partha | Sinha |  |  |  |  |  |  |
| Elizabeth | Oates |  |  |  |  |  |  |
| Gary | Conrad |  |  |  |  |  |  |
| Oscar | Lopez |  |  |  |  |  |  |
| MaryAnn | Oakley |  |  |  |  |  |  |
| Donna | Simpson |  |  |  |  |  |  |
| Anton | Porsteinsson |  |  |  |  |  |  |
| Bonnie | Goldstein |  |  |  |  |  |  |
| Kim | Martin |  |  |  |  |  |  |
| Kelly | Makino |  |  |  |  |  |  |
| M | Saleem Ismail |  |  |  |  |  |  |
| Connie | Brand |  |  |  |  |  |  |
| Ruth | Mulnard |  |  |  |  |  |  |
| Gaby | Thai |  |  |  |  |  |  |
| Catherine | Mc Adams Ortiz |  |  |  |  |  |  |
| Kyle | Womack |  |  |  |  |  |  |
| Dana | Mathews |  |  |  |  |  |  |
| Mary | Quiceno |  |  |  |  |  |  |
| Ramon | Diaz Arrastia |  |  |  |  |  |  |
| Richard | King |  |  |  |  |  |  |

| ***First Name and Middle Initial(s)** | ***Last Name** | ***Suffix (eg, Jr, III)** | **Academic Degrees** | **Institution** | **Location (city, state/province, country)** | **Role or Contribution, eg, chair, principal investigator** | **Group (if more than 1 Group listed in the byline) and/or Subgroup (eg, Steering Committee)** |
| --- | --- | --- | --- | --- | --- | --- | --- |
| Myron | Weiner |  |  |  |  |  |  |
| Kristen | Martin Cook |  |  |  |  |  |  |
| Michael | DeVous |  |  |  |  |  |  |
| Allan | Levey |  |  |  |  |  |  |
| James | Lah |  |  |  |  |  |  |
| Janet | Cellar |  |  |  |  |  |  |
| Jeffrey | Burns |  |  |  |  |  |  |
| Heather | Anderson |  |  |  |  |  |  |
| Russell | Swerdlow |  |  |  |  |  |  |
| Liana | Apostolova |  |  |  |  |  |  |
| Kathleen | Tingus |  |  |  |  |  |  |
| Ellen | Woo |  |  |  |  |  |  |
| Daniel | Silverman |  |  |  |  |  |  |
| Po | Lu |  |  |  |  |  |  |
| George | Bartzokis |  |  |  |  |  |  |
| Neill | Graff Radford |  |  |  |  |  |  |
| Francine | ParfittH |  |  |  |  |  |  |
| Tracy | Kendall |  |  |  |  |  |  |
| Heather | Johnson |  |  |  |  |  |  |
| Martin | Farlow |  |  |  |  |  |  |
| Ann | Marie Hake |  |  |  |  |  |  |
| Brandy | Matthews |  |  |  |  |  |  |
| Scott | Herring |  |  |  |  |  |  |
| Cynthia | Hunt |  |  |  |  |  |  |
| Christopher | van Dyck |  |  |  |  |  |  |
| Richard | Carson |  |  |  |  |  |  |
| Martha G | MacAvoy |  |  |  |  |  |  |
| Howard | Chertkow |  |  |  |  |  |  |
| Howard | Bergman |  |  |  |  |  |  |
| Chris | Hosein |  |  |  |  |  |  |
| Sandra | Black |  |  |  |  |  |  |

| ***First Name and Middle Initial(s)** | ***Last Name** | ***Suffix (eg, Jr, III)** | **Academic Degrees** | **Institution** | **Location (city, state/province, country)** | **Role or Contribution, eg, chair, principal investigator** | **Group (if more than 1 Group listed in the byline) and/or Subgroup (eg, Steering Committee)** |
| --- | --- | --- | --- | --- | --- | --- | --- |
| Bojana | Stefanovic |  |  |  |  |  |  |
| Curtis | Caldwell |  |  |  |  |  |  |
| Ging | Yuek Robin  Hsiung |  |  |  |  |  |  |
| Howard | Feldman |  |  |  |  |  |  |
| Benita | Mudge |  |  |  |  |  |  |
| Michele | Assaly Past |  |  |  |  |  |  |
| Andrew | Kertesz |  |  |  |  |  |  |
| John | Rogers |  |  |  |  |  |  |
| Dick | Trost |  |  |  |  |  |  |
| Charles | Bernick |  |  |  |  |  |  |
| Donna | Munic |  |  |  |  |  |  |
| Diana | Kerwin |  |  |  |  |  |  |
| Marek | Marsel  Mesulam |  |  |  |  |  |  |
| Kristine | Lipowski |  |  |  |  |  |  |
| Chuang | Kuo Wu |  |  |  |  |  |  |
| Nancy | Johnson |  |  |  |  |  |  |
| Carl | Sadowsky |  |  |  |  |  |  |
| Walter | Martinez |  |  |  |  |  |  |
| Teresa | Villena |  |  |  |  |  |  |
| Raymond | Scott Turner |  |  |  |  |  |  |
| Kathleen | Johnson |  |  |  |  |  |  |
| Brigid | Reynolds |  |  |  |  |  |  |
| Reisa A | Sperling |  |  |  |  |  |  |
| Keith A | Johnson |  |  |  |  |  |  |
| Gad | Marshall |  |  |  |  |  |  |
| Meghan | Frey |  |  |  |  |  |  |
| Jerome | Yesavage |  |  |  |  |  |  |
| Joy L | Taylor |  |  |  |  |  |  |
| Barton | Lane |  |  |  |  |  |  |

| ***First Name and Middle Initial(s)** | ***Last Name** | ***Suffix (eg, Jr, III)** | **Academic Degrees** | **Institution** | **Location (city, state/province, country)** | **Role or Contribution, eg, chair, principal investigator** | **Group (if more than 1 Group listed in the byline) and/or Subgroup (eg, Steering Committee)** |
| --- | --- | --- | --- | --- | --- | --- | --- |
| Allyson | Rosen |  |  |  |  |  |  |
| Jared | Tinklenberg |  |  |  |  |  |  |
| Marwan N | Sabbagh |  |  |  |  |  |  |
| Christine M | Belden |  |  |  |  |  |  |
| Sandra A | Jacobson |  |  |  |  |  |  |
| Sherye A | Sirrel |  |  |  |  |  |  |
| Neil | Kowall |  |  |  |  |  |  |
| Ronald | Killiany |  |  |  |  |  |  |
| Andrew E | Budson |  |  |  |  |  |  |
| Alexander | Norbash |  |  |  |  |  |  |
| Patricia | Lynn Johnson |  |  |  |  |  |  |
| Thomas O | Obisesan |  |  |  |  |  |  |
| Saba | Wolday |  |  |  |  |  |  |
| Joanne | Allard |  |  |  |  |  |  |
| Alan | Lerner |  |  |  |  |  |  |
| Paula | Ogrocki |  |  |  |  |  |  |
| Leon | Hudson |  |  |  |  |  |  |
| Evan | Fletcher |  |  |  |  |  |  |
| Owen | Carmichael |  |  |  |  |  |  |
| John | Olichney |  |  |  |  |  |  |
| Charles | DeCarli |  |  |  |  |  |  |
| Smita | Kittur |  |  |  |  |  |  |
| Michael | Borrie |  |  |  |  |  |  |
| T Y | Lee |  |  |  |  |  |  |
| Rob | Bartha |  |  |  |  |  |  |
| Sterling | Johnson |  |  |  |  |  |  |
| Sanjay | Asthana |  |  |  |  |  |  |
| Cynthia M | Carlsson |  |  |  |  |  |  |
| Steven G | G Potkin |  |  |  |  |  |  |
| Adrian | Preda |  |  |  |  |  |  |
| Dana | Nguyen |  |  |  |  |  |  |

| ***First Name and Middle Initial(s)** | ***Last Name** | ***Suffix (eg, Jr, III)** | **Academic Degrees** | **Institution** | **Location (city, state/province, country)** | **Role or Contribution, eg, chair, principal investigator** | **Group (if more than 1 Group listed in the byline) and/or Subgroup (eg, Steering Committee)** |
| --- | --- | --- | --- | --- | --- | --- | --- |
| Pierre | Tariot |  |  |  |  |  |  |
| Adam | Fleisher |  |  |  |  |  |  |
| Stephanie | Reeder |  |  |  |  |  |  |
| Vernice | Bates |  |  |  |  |  |  |
| Horacio | Capote |  |  |  |  |  |  |
| Michelle | Rainka |  |  |  |  |  |  |
| Douglas W | Scharre |  |  |  |  |  |  |
| Maria | Kataki |  |  |  |  |  |  |
| Anahita | Adeli |  |  |  |  |  |  |
| Earl A | Zimmerman |  |  |  |  |  |  |
| Dzintra | Celmins |  |  |  |  |  |  |
| Alice D | Brown |  |  |  |  |  |  |
| Godfrey D | Pearlson |  |  |  |  |  |  |
| Karen | Blank |  |  |  |  |  |  |
| Karen | Anderson |  |  |  |  |  |  |
| Robert B | Santulli |  |  |  |  |  |  |
| Tamar J | Kitzmiller |  |  |  |  |  |  |
| Eben S | Schwartz |  |  |  |  |  |  |
| Kaycee M | SinkS |  |  |  |  |  |  |
| Jeff D | Williamson |  |  |  |  |  |  |
| Pradeep | Garg |  |  |  |  |  |  |
| Franklin | Watkins |  |  |  |  |  |  |
| Brian R | Ott |  |  |  |  |  |  |
| Henry | Querfurth |  |  |  |  |  |  |
| Geoffrey | Tremont |  |  |  |  |  |  |
| Stephen | Salloway |  |  |  |  |  |  |
| Paul | Malloy |  |  |  |  |  |  |
| Stephen | Correia |  |  |  |  |  |  |
| Howard J | Rosen |  |  |  |  |  |  |
| Bruce L | Miller |  |  |  |  |  |  |
| Jacobo | Mintzer |  |  |  |  |  |  |

| ***First Name and Middle Initial(s)** | ***Last Name** | ***Suffix (eg, Jr, III)** | **Academic Degrees** | **Institution** | **Location (city, state/province, country)** | **Role or Contribution, eg, chair, principal investigator** | **Group (if more than 1 Group listed in the byline) and/or Subgroup (eg, Steering Committee)** |
| --- | --- | --- | --- | --- | --- | --- | --- |
| Kenneth | Spicer |  |  |  |  |  |  |
| David | Bachman |  |  |  |  |  |  |
| Elizabether | Finger |  |  |  |  |  |  |
| Stephen | Pasternak |  |  |  |  |  |  |
| Irina | Rachinsky |  |  |  |  |  |  |
| John | Rogers |  |  |  |  |  |  |
| Andrew | Kertesz |  |  |  |  |  |  |
| Dick | Drost |  |  |  |  |  |  |
| Nunzio | Pomara |  |  |  |  |  |  |
| Raymundo | Hernando |  |  |  |  |  |  |
| Antero | Sarrael |  |  |  |  |  |  |
| Susan K | Schultz |  |  |  |  |  |  |
| Laura L | Boles Ponto |  |  |  |  |  |  |
| Hyungsub | Shim |  |  |  |  |  |  |
| Karen | Elizabeth Smith |  |  |  |  |  |  |
| Norman | Relkin |  |  |  |  |  |  |
| Gloria | Chaing |  |  |  |  |  |  |
| Lisa | Raudin |  |  |  |  |  |  |
| Amanda | Smith |  |  |  |  |  |  |
| Kristin | Fargher |  |  |  |  |  |  |
| Balebail | Ashok Raj |  |  |  |  |  |  |
